# Intra‐household double burden of overweight/obesity and anaemia: Evidence from 49 low‐and middle‐income countries

**DOI:** 10.1111/mcn.13298

**Published:** 2021-12-22

**Authors:** Ana Irache, Paramjit Gill, Rishi Caleyachetty

**Affiliations:** ^1^ Warwick Centre for Global Health, Division of Health Sciences, Warwick Medical School University of Warwick Coventry UK; ^2^ Nuffield Department of Medicine University of Oxford Oxford UK

**Keywords:** anaemia, double burden of malnutrition, inequalities, low‐and middle‐income countries, obesity, overweight

## Abstract

Globally, overweight/obesity is rising rapidly while anaemia persists. Nevertheless, evidence on their coexistence at the household level remains limited. Using data from the Demographic and Health Surveys, we quantified the magnitude, distribution and inequalities (i.e., estimates by wealth, education level and residence) in the intra‐household double burden (DBM) of overweight/obesity and anaemia among mothers and their children living in 49 low‐ and middle‐income countries (LMICs). The pooled prevalence of total intra‐household DBM was 17.2% (95% confidence interval [CI]: 15.6, 18.8); 16.2% (95% CI: 14.6, 17.9) for mothers with overweight/obesity and children with anaemia; and 2.8% (95% CI: 2.5, 3.1) for mothers with anaemia and children with overweight/obesity. South Africa had the highest prevalence of total DBM at the household level, affecting almost one in three households. Households with mothers with overweight/obesity and children with anaemia followed an inverse social gradient, with higher estimates found in the richest quintile, highest maternal education level and in urban areas; although with some variation across regions. The opposite was observed for mothers with anaemia and children with overweight/obesity. The largest inequality gaps were found for mothers with overweight/obesity and children with anaemia in Togo by household wealth (29.3%‐points; *p* < 0.001), in Ghana by maternal education level (28.0%‐points; *p* = 0.001) and in Niger by area of residence (25.2%‐points; *p* < 0.001). Although double‐duty actions might help accelerate action towards reducing malnutrition in all its forms, a comprehensive assessment of the causes of anaemia is first warranted to design effective country‐specific programmes.

## Key messages

- Using anthropometric and anaemia data from 49 low‐ and middle‐income countries, we found an overall pooled prevalence of total intra‐household double burden of malnutrition of 17.2%, with South Africa bearing the highest burden.
- The prevalence of mothers with overweight/obesity and children with anaemia ranged from 3.1% (Ethiopia) to 42.2% (South Africa), and was higher in the richest quintile, highest maternal education level and in urban areas.
- Mothers with anaemia and children with overweight/obesity was low (<9.0% in all countries).

## 1 INTRODUCTION

In low‐and middle‐income countries (LMICs) women and children are disproportionally affected by malnutrition, where forms of undernutrition coexist with overweight/obesity within individuals, households and populations across the life‐course (Development Initiatives, 2020; Popkin et al., 2020). Overlapping forms of malnutrition referred as the double burden of malnutrition (DBM), are the direct consequence of increases in the prevalence of overweight/obesity over the last decades, as a result of the ongoing nutrition transition and food system transformations in LMICs, coupled up with persistent forms of undernutrition (including micronutrient deficiencies; Popkin et al., 2020; WHO, 2017).

Overweight/obesity is rapidly rising globally, particularly in low‐income countries and urban areas, with adult women bearing the highest burden of obesity (15.1%) when compared with males (11.1%; Amugsi et al., 2017; Development Initiatives, 2020; Jiwani et al., 2020). Excess body fat increases the risk of developing noncommunicable diseases (e.g., diabetes and cardiovascular disease) and is associated with maternal morbidity, preterm birth and infant mortality (Abdullah et al., 2011; Black et al., 2013). Among children under‐5, the prevalence of overweight/obesity has also risen from 4.9% in 2000 to 5.6% in 2019 (UNICEF/WHO/The World Bank, 2020). The presence of overweight/obesity early in life can predispose individuals to chronic diseases and increased risk of mortality in adulthood (Reilly & Kelly, 2011). Anaemia currently affects 32.8% of women of reproductive age (15–49 years old) and 43.0% of children under‐5 (Balarajan et al., 2011; Development Initiatives, 2020), causing physical and cognitive impairments, fatigue and low productivity (Haas & Brownlie, 2001; WHO, 2014). Maternal anaemia can also contribute to maternal deaths and low‐birthweight (Black et al., 2013; WHO, 2014). The most common causes of anaemia include nutritional deficiencies (e.g., iron, folate or vitamins B and A), and as such, it is often used as a proxy for micronutrient deficiencies in the absence of micronutrient data (Cameron & Neufel, 2011; WHO, 2014). However, the aetiology of anaemia is complex and context‐specific (Chaparro & Suchdev, 2019). Other identified risk factors for anaemia include infectious diseases, inflammation, haemoglobinopathies or parasitic infections (Chaparro & Suchdev, 2019).

Progress to reduce malnutrition has been slow and unequal. In LMICs, forms of undernutrition among children under‐5 continue to disproportionally affect those living in the poorest households, rural areas and with less educated mothers; whereas the reverse is observed for overweight (Development Initiatives, 2020). Similarly, among women, overweight is more prevalent in the richest households and urban areas, with less pronounced inequalities by education level; meanwhile, anaemia continues to be a threat to all women regardless of socioeconomic background (Jiwani et al., 2020). Nevertheless, recent evidence suggests a shift of the adult burden of overweight and obesity towards the poor, as well as rapid increases in BMI among rural residents (Jiwani et al., 2019; NCD Risk Factor Collaboration, 2019; Templin et al., 2019). As a result, no country is on track to achieve both, the 2025 Global Nutrition Target of anaemia among women of reproductive age and the NCD Target of adult obesity; meanwhile, 53 countries are on course to meet the under‐5 overweight Global Nutrition Target and there is no target for childhood anaemia (Development Initiatives, 2020). Identifying subgroups for which the DBM is highest is particularly relevant to develop more tailored nutritional policies and interventions that targets those who are most in need and accelerate progress towards the established nutritional targets.

The co‐occurrence of overweight/obesity and anaemia has been previously documented at the individual and population levels (Davis et al., 2020; Engle‐Stone et al., 2020; Irache et al., 2021; Williams et al., 2020). At the household level, a large body of evidence exists for the coexistence of overweight/obesity and stunting or wasting, including the Lancet series on the DBM (Biswas et al., 2020; Davis et al., 2020; Kroker‐Lobos et al., 2014; Popkin et al., 2020). Yet, the extent to which overweight/obesity and anaemia coexist at this level across LMICs, as well as which subgroups of the population are at the highest risk, remains poorly defined, with no global estimates published. Studies from individual countries point to a high prevalence of intra‐household co‐occurrence of overweight/obesity among mothers and anaemia in children (Sassi et al., 2018; Varghese & Stein, 2019). To address these gaps in knowledge, we aimed to examine the magnitude, distribution and inequalities in the intra‐household double burden of overweight/obesity and anaemia by household wealth, education level and area of residence, among mothers and their children under‐5 living in LMICs.

## 2 METHODS

### 2.1 Data sources and study population

We used the most recent Demographic and Health Surveys (DHS), January 2000–2019, from all LMICs with available anthropometric and haemoglobin level measures for women of reproductive age (15–49 years old) and their children under‐5. The DHS are comparable nationally representative household surveys from over 90 countries, undertaken approximately every 5 years. These surveys contain data on the population, health and nutrition, including measured weight and height variables and haemoglobin levels for the diagnosis of anaemia among mothers and their children. In all countries, DHS follows standardised procedures (e.g., survey instruments and data collection methods). Complete descriptions of country DHS sampling, questionnaire validation, data collection methods and data validation procedures are published elsewhere (Croft et al., 2018). Before conducting any questionnaire or biomarker tests, informed consent was taken from all participants.

Mothers who were pregnant or who have given birth in the 2 months preceding data collection were excluded from the study, due to weight gain during pregnancy, and following DHS guidelines (Croft et al., 2018). Participants with missing anthropometric measures or haemoglobin levels (e.g., missing values or data not recorded), and those with biologically implausible values were also excluded from the analytic sample. For women of reproductive age, height and weight outside of the ranges 100–220 cm and 20–220 kg, respectively, were set as missing values, as were BMI‐for‐age *z*‐scores < −5 and >5 for children under‐5 (Croft et al., 2018). Haemoglobin concentrations outside of the 4.0–18.0 g/dl range were also considered as biologically implausible values (Sullivan et al., 2008). We used the individual's record (coded as ‘IR’) data sets for each country which contained information matched for mothers aged 15–49 years old and their children (0–59 months) living in one household.

### 2.2 Anthropometry and anaemia measures

Trained personnel weighed and measured participants using a SECA digital scale and Shorr Productions measuring board. Diagnosis of anaemia was confirmed using HemoCue® 201+ or the 301+ system, a portable haemoglobin analyser that measures haemoglobin concentration levels in capillary blood.

To define overweight/obesity among mothers, we used the Quetelex index for adult women (20–49 years old; WHO, 2020), and the WHO 2007 growth standards (5–19 years) for adolescent girls (15–19 years old; WHO, 2007). According to this, adult women were categorised as having overweight/obesity if their body mass index (BMI) was ≥25.0 kg/m^2^; whereas among adolescent girls, overweight/obesity was defined as BMI‐for‐age *z*‐score > 1 SD above from the median of the reference population. For children under‐5 (0–59 months), we used the WHO 2006 child growth standards, and overweight/obesity was defined as a BMI‐for age *z*‐score > 2 SD (Furlong et al., 2016; WHO, 2006).

Anaemia in mothers (15–49 years old) was defined as haemoglobin concentration levels adjusted for altitude and smoking (which are known factors to increase haemoglobin concentrations) <12.0 and <11.0 g/dl in children (6–59 months; Pullum et al., 2017; Sharma et al., 2019). For anaemia only, the analysis among children was restricted to those aged 6–59 months, as data from infants younger than 6 months are not collected for presenting higher haemoglobin levels which may distort the indication of the prevalence of anaemia (Croft et al., 2018; WHO, 2001).

### 2.3 Defining the intra‐household DBM

Following the WHO (2017) definition, we defined the intra‐household DBM as multiple family members (i.e., mothers aged 15–49 years old living with their children under‐5) affected by different forms of malnutrition (i.e., overweight/obesity and anaemia). Therefore, we identified two main forms of household‐level DBM: (i) a mother has overweight/obesity and at least one of her children has anaemia, or (ii) a mother has anaemia and at least one of her children has overweight/obesity.

Using the DHS individual records, each mother represented one household with up to six children under‐5. We first created binary variables to identify whether for each household there were at least one child living with either anaemia or overweight/obesity separately. Then, we created two binary variables, one for mothers with overweight/obesity and children with anaemia and a second one for mothers with anaemia and children with overweight/obesity. Lastly, we also calculated the total intra‐household DBM, defined as a household presenting either one of the two identified forms of household‐level DBM, or both.

### 2.4 Sociodemographic measures

The sociodemographic characteristics to explore the distribution and inequalities of the intra‐household DBM were wealth, education and residence. DHS measures household wealth as a composite measure of household assets (e.g., bicycles, cars or radios) and characteristics (e.g., flooring material, drinking water source or type of toilet facility). Household wealth was further categorised into five quintiles (Q1: poorest; Q2: poorer; Q3: middle; Q4: richer; Q5: richest) within each country (Rutsein & Johnson, 2004). Maternal education level was assessed by self‐report of the mother's completed educational level and divided into four levels (E1: no education; E2: primary education; E3: secondary education; E4 higher education). The household's area of residence was defined according to country‐specific definitions and categorised as urban or rural.

### 2.5 Statistical analysis

We first calculated prevalence estimates and 95% confidence intervals (CIs) of the two different combinations of intra‐household DBM, as well as the total DBM burden, for every country. Stratified estimates by household wealth quintile, maternal education level and area of residence were also calculated for the first two combinations of DBM. Following DHS guidelines, we excluded prevalence estimates for which the sample size of the subgroup of households was lower than 25 observations (Croft et al., 2018).

Meta‐analyses were performed (Stata command ‘*metaprop*’) to estimate the pooled prevalence and 95% CIs of each form of intra‐household DBM overall and by WHO region (i.e., African, Eastern Mediterranean, European, Americas, Southeast Asian and Western Pacific), using a random‐effects model (Barendregt et al., 2013; Nyaga et al., 2014). The regional pooled prevalence for the Western Pacific could not be generated, as it only had one country (Cambodia) with available data.

To display and further understand inequalities in the distribution of the DBM, we measured inequality gaps, defined as the absolute difference in percentage points between the intra‐household DBM prevalence in the two most extreme opposite groups across each socioeconomic measure: richest versus poorest wealth quintile (Q5–Q1), highest versus lowest maternal education level (E4–E1) and urban versus rural (urban–rural). A positive gap value depicts a higher prevalence of intra‐household DBM in the richest quintile (Q5), highest maternal education level (E4) and urban areas; whereas a negative gap value depicts a higher prevalence in the poorest quintile (Q1), lowest education level (E1) and in rural areas. We calculated whether differences observed across the different groups were significant (*p* < 0.05), or rather due to chance, through *χ* ^2^ tests and tests for trend.

All analyses were conducted on Stata version V.16.0 (StataCorp). We used Stata's survey estimation procedures (‘*svy*’ command) throughout the analyses to take into account the complex survey weights and sampling designs of DHS surveys.

## 3 RESULTS

### 3.1 Characteristics of surveys and households

Overall, 49 LMICs had a DHS between 2005 and 2018 with available anthropometric and anaemia measures for both, mothers and their children under‐5. By WHO region, per total number of LMICs included in the study (*n* = 49), 59.2% (*n* = 29) were from the African region, 4.1% (*n* = 2) from the Eastern Mediterranean region, 12.2% (*n* = 6) from the European region, 12.2% (*n* = 6) from the Americas region, 10.2% (*n* = 5) from the Southeast Asian region and 2.1% (*n* = 1) from the Western Pacific region. The total analytical sample size comprised 311,604 households (encompassing mothers and their children under‐5) with 272,039 households for mothers with overweight/obesity and children with anaemia, and 286,414 households for mothers with anaemia and children with overweight/obesity. The total household‐level DBM was calculated in a total sample of 292,977 households.

Characteristics of households included in the study are provided for each country in Tables S1 and S2. The proportion of households in the poorest group (Q1) ranged from 17.0% (Egypt) to 25.3% (Guyana), and from 12.3% (Namibia) to 22.8% (Armenia) for households in the richest group (Q5; Table S1). Niger had the highest prevalence of households with mothers with no education (85.0%); whereas Armenia had the lowest prevalence of uneducated mothers (0.0%) and the highest prevalence of higher maternal education (54.6%; Table S1). The proportion of urban households ranged from 9.4% in Burundi to 85.9% in Gabon (Table S1). Individual forms of malnutrition were higher in Egypt for households with mothers with overweight/obesity (80.1%) and households where at least one child was affected by overweight/obesity (21.5%); in Yemen (71.5%) for households with mothers with anaemia; and in Burkina Faso (90.6%) for households where at least one child had anaemia (Table S2).

### 3.2 Total DBM at the household level

The pooled prevalence of total intra‐household DBM was 17.2% (95% CI: 15.6, 18.8; *I* ^2^: 99.2%), ranging from 4.1% in Ethiopia to 38.7% in South Africa (Table S3). The pooled regional prevalence ranged from 12.6% (95% CI: 8.5, 16.7) in the Southeast Asian region to 25.3% (95% CI: 24.1, 26.4) in the Eastern Mediterranean region.

### 3.3 Households with overweight/obesity among mothers and anaemia among children

**Figure 1:**
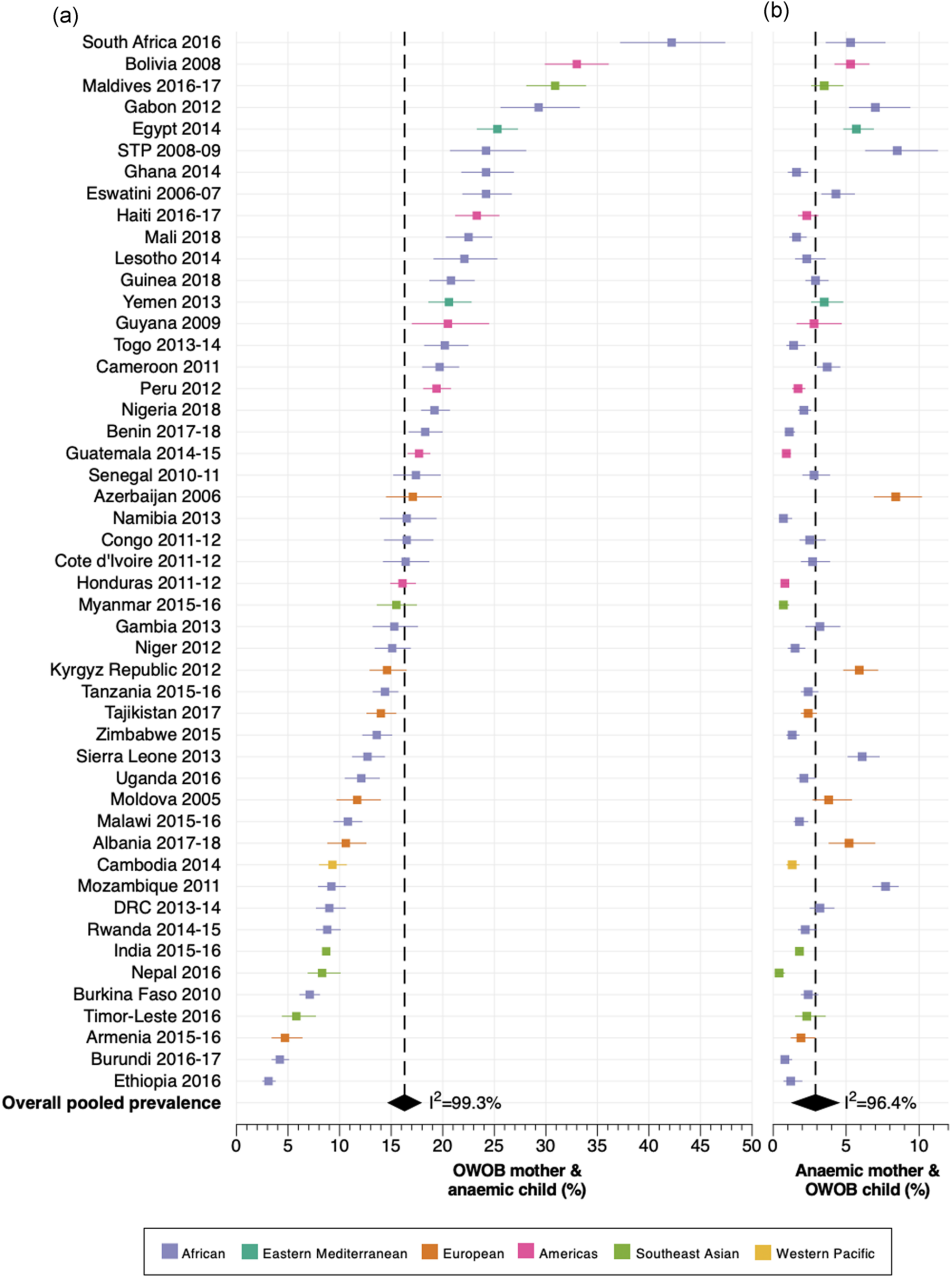
Country‐level magnitude of the intra‐household double burden: (A) households with overweight/obesity among mothers and anaemia among children and (B) households with anaemia among mothers and overweight/obesity among children. DRC, Democratic Republic of the Congo; OWOB, overweight/obesity; STP, Sao Tome and Principe

The pooled prevalence of mothers with overweight/obesity and children with anaemia was 16.2% (95% CI: 14.6, 17.9; *I* ^2^: 99.3%), ranging from 3.1% in Ethiopia to 42.2% in South Africa (Figure 1 and Table S3). The pooled regional prevalence ranged from 12.1% (95% CI: 8.4, 15.8) in the European region to 24.1% (95% CI: 22.9, 25.3) in the Eastern Mediterranean region.

**Figure 2:**
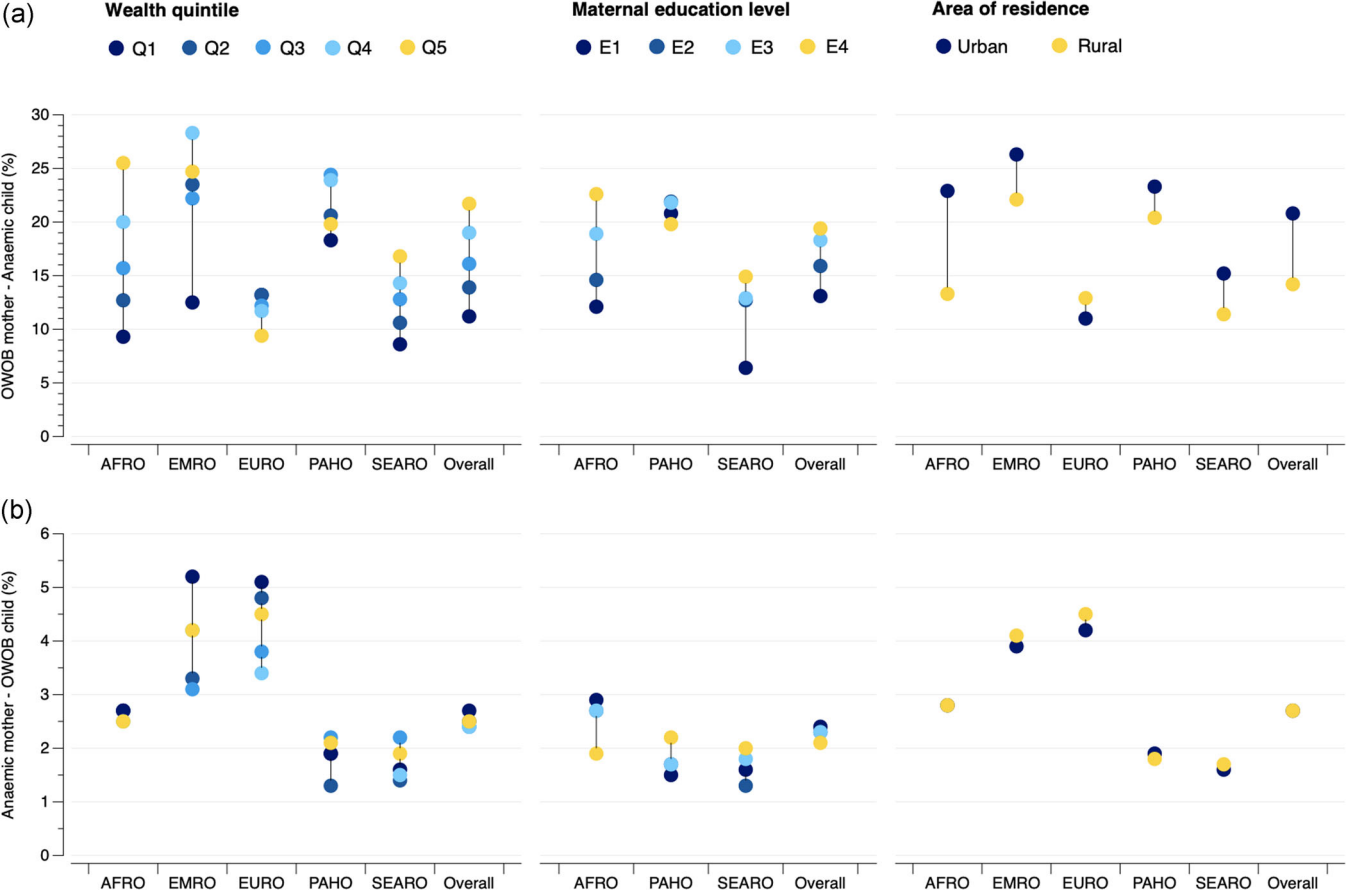
Distribution of the intra‐household DBM by household wealth quintile, maternal education level and area of residence across WHO regions and overall: (a) households with overweight/obesity among mothers and anaemia among children and (b) households with anaemia among mothers and overweight/obesity among children. Wealth quintiles: Q1 (poorest), Q2 (poorer), Q3 (middle), Q4 (richer), Q5 (richest). Maternal education levels: E1 (no education), E2 (primary education), E3 (secondary education), E4 (higher education). The EMRO and EURO regions are missing for maternal education level, as 1/2 and 5/6 countries, respectively, had sample sizes below 25 observations for one or two education levels; and thus, the regional pooled prevalence could not be calculated. All countries with sample sizes above 25 observations for the five wealth quintiles, four maternal education levels and urban/rural areas were included in the calculation of the overall pooled prevalence estimates. AFRO, African region; EMRO, Eastern Mediterranean region; EURO, European region; OWOB, overweight/obesity; PAHO, Americas region; SEARO, Southeast Asian region

The distribution of the magnitude of this form of intra‐household DBM for all LMICs is presented in Tables S4–S6. Overall, the highest prevalence of mothers with overweight/obesity and children with anaemia was found in the richest household wealth quintile (21.7%), highest maternal education level (19.4%), and in urban areas (20.8%); whereas, the lowest prevalence corresponded with the lowest household wealth quintile (11.2%), lowest maternal education level (13.1%) and rural areas (14.2%; Figure 2a). By WHO region, the inverse was observed in the European region, where the prevalence was highest in the first and second wealth quintiles, and in rural areas; while the lowest prevalence was found in households from the fifth wealth quintile and those located in urban areas. Moreover, in the Americas region, the prevalence of DBM was highest in the third and fourth household wealth quintiles and lowest in the fourth maternal education level. In the Eastern Mediterranean region, the prevalence of DBM was also highest in the fourth wealth quintile (Figure 2a).

**Figure 3:**
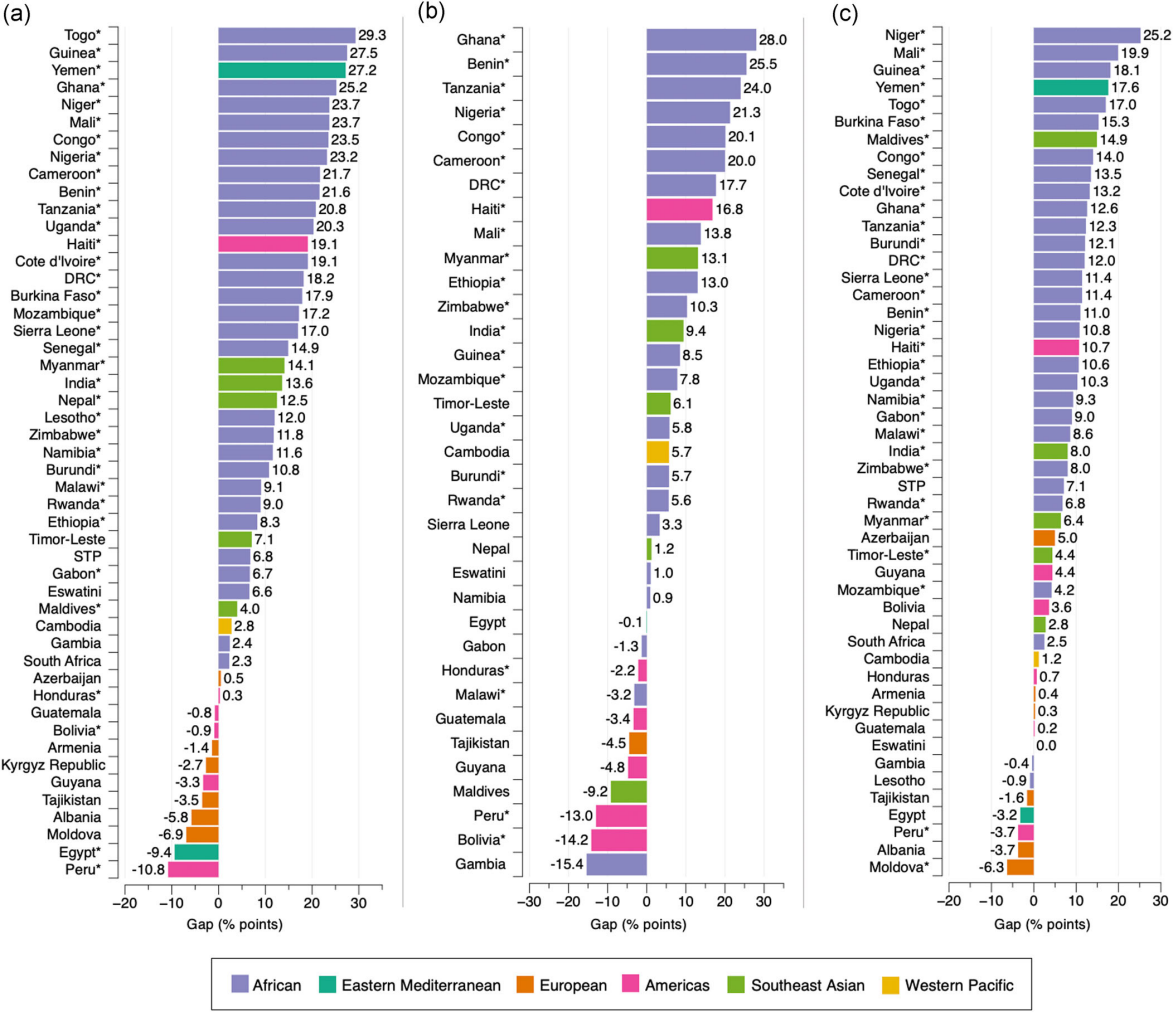
Absolute gap difference of households with overweight/obesity among mothers and anaemia among children by wealth quintile (a), maternal education level (b) and area of residence (c). Positive values mean that intra‐household DBM is more prevalent in the richest quintile (Q5), highest maternal education level (E4) and in urban areas when compared with the poorest quintile (Q1), lowest maternal education level (E1) and rural areas. Negative values mean the opposite. *p < 0.05. Note that in figure (b) countries with a sample size <25 observations for E1 or E4 were excluded. DRC, Democratic Republic of the Congo; STP, Sao Tome and Principe

Figure 3 shows the absolute inequality of the prevalence of households with overweight/obesity among mothers and anaemia among children by the three socioeconomic measures and by LMICs. Large inequalities were observed in the distribution of this combination, particularly by household wealth, with 12 countries showing a difference higher than 20 percentage points between the fifth and first wealth quintiles (Figure 3a). The largest gaps were observed in Togo, with a 29.3 percentage‐point difference (*p* < 0.001) in intra‐household DBM prevalence by household wealth (Q1, 7.1%; Q5, 36.4%); Ghana, with a 28.0 percentage‐point difference (*p* = 0.001) by maternal education level (E1, 15.8%; E4, 43.8%); and in Niger, with a 25.2 percentage‐point difference (*p* < .001) by area of residence (urban, 36.4%; rural, 11.2%).

Gaps were positive in 79.6% (39/49), 68.6% (24/35) and 83.7% (41/49) of countries by household wealth quintile, maternal education level and area of residence, respectively (Figure 3). This indicates that for most LMICs, households with overweight/obesity among mothers and anaemia among children were most commonly found among the richest wealth quintile, highest maternal education level and in urban areas, when compared with the poorest wealth quintile, lowest maternal education level and rural areas. Negative gaps depicting the opposite (i.e., higher prevalence of intra‐household DBM in the poorest wealth quintile, lowest maternal education level and in rural areas), were observed in a low number of LMICs: 20.4% (10/49) by household wealth quintile, 31.4% (11/35) by maternal education level, and 14.3% (7/49) by area of residence. For one African country (Eswatini), the prevalence of intra‐household DBM was the same in urban and rural areas (24.2%), and thus, the inequality gap was 0.0 percentage points by area of residence (Figure 3c).

Differences observed across groups were statistically significant in 71.4% (35/49), 62.9% (22/35) and 65.3% (32/49) of countries by household wealth, maternal education level and area of residence, respectively (Figure 3 and Tables S4–S6).

### 3.4 Households with anaemia among mothers and overweight/obesity among children

The pooled prevalence of mothers with anaemia and children with overweight/obesity was 2.8% (95% CI: 2.5, 3.1; *I* ^2^: 96.4%), ranging from 0.4% in Nepal to 8.5% in Sao Tome and Principe (Figure 1). The pooled regional prevalence was lowest in the Southeast Asian region, with a 1.7% (95% CI: 0.8, 2.6) prevalence of intra‐household DBM, and highest in the European region, with a 4.4% (95% CI: 2.9, 6.0) prevalence (Table S3).

Overall, the prevalence of mothers with anaemia and children with overweight/obesity was significantly lower than mothers with overweight/obesity and children with anaemia, with an overall difference of 13.4 percentage points between both intra‐household DBM forms (Figure 1). Only in six countries, the prevalence of DBM was similar for both forms (difference < 5 percentage points), although higher for mothers with overweight/obesity and children with anaemia, including in Burkina Faso (7.1% vs. 2.4%), Burundi (4.2% vs. 0.8%), Ethiopia (3.1% vs. 1.2%), Mozambique (9.2% vs. 7.7%), Armenia (4.7% vs. 1.9%) and Timor‐Leste (5.8% vs. 2.3%).

**Figure 4:**
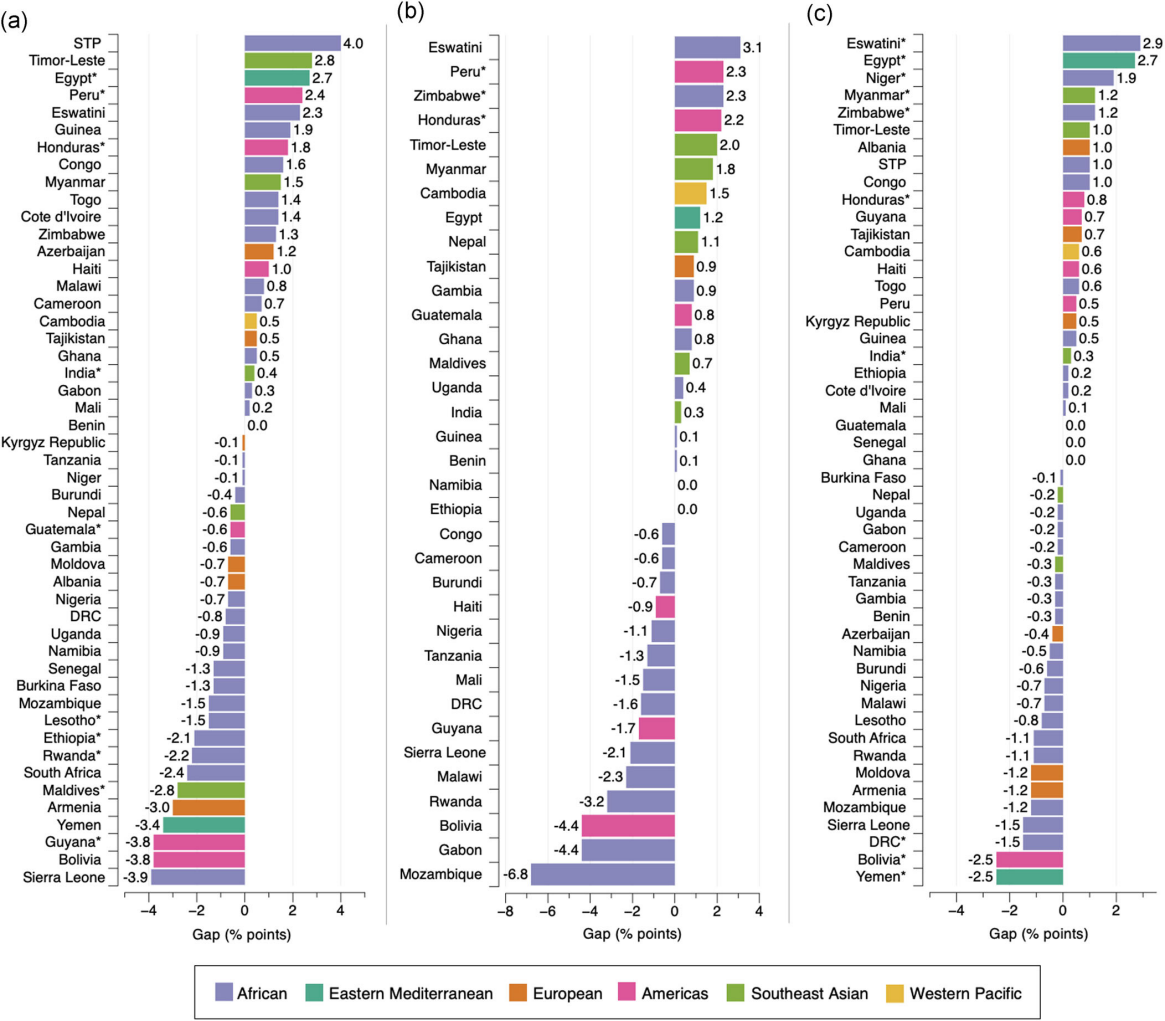
Absolute gap difference of households with anaemia among mothers and overweight/obesity among children by wealth quintile (a), maternal education level (b) and area of residence (c). Positive values mean that intra‐household DBM is more prevalent in the richest quintile (Q5), highest maternal education level (E4) and in urban areas when compared with the poorest quintile (Q1), lowest maternal education level (E1) and rural areas. Negative values mean the opposite. *p < 0.05. Note that in figure (b) countries with a sample size <25 observations for E1 or E4 were excluded. DRC, Democratic Republic of the Congo; STP, Sao Tome and Principe

The distribution of mothers with anaemia and children with overweight/obesity differed from that of mothers with overweight/obesity and children with anaemia; and differences in prevalence across groups was minimal, although with some exceptions (Figure 2 and Tables S7–S9). Overall, the highest prevalence of mothers with anaemia and children with overweight/obesity was found in the poorest household wealth quintile (2.7%) and lowest maternal education level (2.4%); whereas the lowest prevalence was observed in the third and fourth household wealth quintile (2.5%)and the highest maternal education level (2.1%) (Figure 2b). By area of residence, both urban and rural areas had an overall 2.7% prevalence of this form of intra‐household DBM; although the prevalence was slightly higher among rural residents in the Eastern Mediterranean, European and Southeast Asian regions (Figure 2b). A distinct pattern was found in the Americas region by the three socioeconomic measures (i.e., highest prevalence in the third wealth quintile, highest maternal education level and in urban areas), and in the Southeast Asian region by household wealth and maternal education (Figure 2b). The widths of inequality gaps were largely less pronounced than those for mothers with overweight/obesity and children with anaemia, with only 11 instances where gaps in the prevalence of mothers with anaemia and children with overweight/obesity were equal or greater than 3.0 percentage points (Figure 4a–c). The largest gaps were found in Sao Tome and Principe, with a 4.0 percentage‐point difference (*p* = 0.527) in intra‐household DBM by household wealth (Q1, 8.5%; Q5, 12.5%); Mozambique, with a −6.8 percentage‐point difference (*p* = 0.189) by maternal education level (E1, 8.4%; E4, 1.6%); and Eswatini, with a 2.9 percentage‐point difference (*p* = 0.035) by area of residence (urban, 6.7%; rural, 3.8%).

Gaps were positive in 44.9% (22/49) of countries by household wealth and area of residence, and in 51.4% (18/35) of countries by maternal education level (Figure 4); while gaps were negative in 53.1% (26/49), 42.9% (15/35), 45.0% (24/49) of countries by household wealth, maternal education level and area of residence, respectively. In six instances, the inequality gap in the prevalence of intra‐household DBM was 0.0 percentage points, meaning that the prevalence of mothers with anaemia and children with overweight/obesity was the same in the richest and poorest wealth quintiles in Benin (0.8%); the most and least educated in Namibia (0.0%) and Ethiopia (1.0%); and among urban and rural residents in Guatemala (0.9%), Senegal (2.8%) and Ghana (1.6%; Figure 4a–c).

Differences in the prevalence of mothers with anaemia and children with overweight/obesity observed across groups were statistically significant in 10/49, 3/35 and 10/49 of countries by household wealth, maternal education level and area of residence, respectively (Figure 4 and Tables S7–S9).

## 4 DISCUSSION

We quantified the magnitude, distribution and inequalities of the co‐occurrence of overweight/obesity and anaemia at the household level using nationally representative DHS samples across 49 LMICs from 2005 to 2018. Our results show that almost 2 in 10 households presented a form of intra‐household DBM, with South Africa bearing the highest burden (38.7%). Households with overweight/obesity among mothers and anaemia among children was the leading form of intra‐household DBM (16.2%), when compared to households with anaemia among mothers and overweight/obesity among children (2.8%). Important variations in the prevalence of DBM were observed across and within countries, particularly for mothers with overweight/obesity and children with anaemia.

To the best of our knowledge, no previous studies have comprehensively explored the prevalence of overweight/obesity and anaemia among mothers and their children under‐5 at the household level across LMICs, as well as its distribution and inequalities by socioeconomic measures (i.e., household wealth, education level and area of residence). A systematic review quantifying the frequency of the different DBM operational definitions, only found one article published before July 2017 examining the co‐occurrence of overweight/obesity and anaemia at the household level (Davis et al., 2020). Sassi et al. (2018) estimated the magnitude of mothers with overweight and children with anaemia living in Tunisia to be 24.4%, which is similar to our pooled prevalence for households with overweight/obesity among mothers and anaemia among children for the Eastern Mediterranean region (24.1%). Christian and Dake (2021) examined the household‐level double and triple burden of malnutrition in Sub‐Saharan African countries. Our study used different definitions of intra‐household DBM, and therefore, our estimates are not directly comparable.

Our findings complement The Lancet Series on the DBM that quantified the coexistence of overweight/obesity and stunting, wasting or thinness at the household level. Popkin et al. (2020) found the combination women with overweight and children with stunting to be the most prevalent of all three possible scenarios (i.e., mothers with overweight and children with stunting; mothers with overweight and children with wasting; and mothers with thinness and children with overweight), ranging from 1.1% in Vietnam to 24.3% in Guatemala. Similarly, we found the highest prevalence of intra‐household DBM in the combination with overweight/obesity among mothers and the form of undernutrition (i.e., anaemia) among children, ranging from 3.1% in Ethiopia to 42.2% in South Africa. This is not surprising as overweight/obesity remains low among children under‐5, while it is rising rapidly among women of reproductive age in most LMICs (Development Initiatives, 2020). A hypothesis that has been put forward for this coexistence is poor quality diets characterised by the increased availability and consumption of ultra‐processed foods in LMICs, which are energy‐dense, but poor in vitamins and minerals, associated with excess weight and, possibly anaemia (Chen et al., 2020; Monteiro et al., 2011, 2013; Pagliai et al., 2021). Another plausible explanation for a high coexistence of intra‐household DBM could be that maternal overweight/obesity increases the risk of childhood anaemia. Multiple studies suggest that obesity during pregnancy can impair both, maternal and neonatal iron status (Jones et al., 2016; Phillips et al., 2014; Wawer et al., 2021). Moreover, maternal obesity increases the risk of fetal macrosomia, which in turn, may lead to inflammation, a rise in hepcidin levels, and over time, result in anaemia of inflammation (Ovesen et al., 2011; Wawer et al., 2021).

The co‐occurrence of overweight/obesity and anaemia at the household level was unequally distributed, particularly the form with overweight/obesity among mothers and anaemia among children, by household wealth, maternal education level and area of residence. The latter followed an inverse social gradient, emulating the distribution of maternal overweight/obesity and maternal concurrent overweight/obesity and anaemia in LMICs, with overall higher estimates in the richest quintile, highest maternal education level and urban areas (Hossain et al., 2020; Irache et al., 2021; Jiwani et al., 2019, 2020; Matos et al., 2020). The different distribution patterns observed in the European and the Americas region for mothers with overweight/obesity and children with anaemia could be a reflection of changing trends documented in the prevalence of overweight/obesity towards the poorest groups and those living in rural areas (Jiwani et al., 2019; Monteiro et al., 2004; NCD Risk Factor Collaboration, 2019). If this was true, we could expect similar changes in patterns in the distribution of the intra‐household DBM in the other regions as countries go through the different stages of the nutrition transition. Moreover, our analyses of absolute inequality for mothers with overweight/obesity and children with anaemia showed large gaps by the three socioeconomic measures for most African countries and in Yemen by household wealth. In one‐quarter of all LMICs included in the analysis, the inequality gap in DBM was higher than 20.0 percentage points between the richest and poorest households. For example, in Togo, where the gap was highest (29.3 percentage points), the prevalence of mothers with overweight/obesity and children with anaemia ranged from 7.1% in the first wealth quintile to 36.4% in the fifth quintile. Nevertheless, inequalities were low in South Africa, where the prevalence of this form of intra‐household DBM was the highest (42.2%), showing estimates >36.0% for all household wealth quintiles, maternal education levels, and in urban and rural areas. This points to the need for context‐specific interventions that responds to the specific nutritional needs of sub‐populations within individual LMICs.

We observed negligible differences in the prevalence of mothers with anaemia and children with overweight/obesity by the different sociodemographic measures, which is likely a result of lower proportions of overweight/obesity among children under‐5, even though maternal anaemia is prevalent in LMICs. The overall inequality gap in this form of DBM was below 0.5 percentage points by household wealth (Q1: 2.7%; Q5: 2.6%), maternal education (E1: 2.5% vs. E4: 2.2%) and place of residence (urban: 2.7% vs. rural: 2.8%).

Our study is not without limitations. First, we used anaemia given that DHS surveys do not collect individual micronutrient deficiencies for the majority of LMICs. The proportion of anaemia among women of reproductive age and children attributed to iron deficiency is approximately 71.0% and 50.0% in countries with a low infection burden, respectively (Engle‐Stone et al., 2017; Wirth et al., 2017). This proportion drops to 35.1% among women and remains at 58.0% among children under‐5 in countries with a high infection burden (Engle‐Stone et al., 2017; Wirth et al., 2017). Second, evidence has shown substantial differences in the estimation of anaemia depending on the method used to measure haemoglobin levels (Hruschka et al., 2020). DHS follows similar standardised procedures to collect haemoglobin levels through capillary blood across countries. Differences between our estimates and those of similar studies using other data sources could be explained by the method used for haemoglobin assessment. Other factors that can lead to variability in the prevalence of anaemia include environmental factors, HemoCue® model, or seasonality (Hruschka et al., 2020). Third, we included the most recent DHS from all countries with available anthropometry and anaemia status among mothers and their children to the analysis; however, most countries were from the African region (*n* = 29). Other WHO regions are likely to be underrepresented (i.e., the Eastern Mediterranean region [*n* = 2] or the Western Pacific [*n* = 1]), which limits the generalisability of our results. Fourth, the sample size for the different subgroups in the stratified analyses by maternal education level were lower than 25 observations in 13 out of the 49 LMICs included. Therefore, we could not calculate the inequality gap for those countries, as well as the pooled regional estimate for the European region. Likewise, we were not able to calculate the pooled estimate for the Eastern Mediterranean region as Yemen did not have data on maternal education level. Fifth, we used the most recent DHS surveys available for each country; however, these ranged from 2005 (Moldova) to 2018 (Guinea, Mali, and Nigeria). Therefore, country‐level estimates from older surveys might not reflect the current intra‐household DBM magnitude. Despite these limitations, our study has several strengths. To the best of our knowledge, this is the largest study providing estimates on the intra‐household double burden of overweight/obesity and anaemia across LMICs (*n* = 49) and WHO regions, as well as exploring the distribution and inequalities by three socioeconomic measures. Moreover, we were able to analyse overall large sample sizes from nationally representative surveys.

The study findings may support a better understanding on the intra‐household double burden of overweight/obesity and anaemia. Historically, the national nutrition policies of LMICs have mainly focused on childhood undernutrition, while ignoring the rapidly rising problem of overweight/obesity in these countries. This siloed approach has resulted in harmful unintended consequences. For example, a nutrition programme implemented in Guatemala that provided fortified food supplements in the first 1000 days to mothers and children, successfully reduced childhood stunting, but also led to greater maternal weight retention (Leroy et al., 2019). Hawkes et al. (2020) have proposed a list of 10 double‐duty actions, which aim to simultaneously tackle both undernutrition and overweight/obesity, including the promotion of adequate nutrition early in life (e.g., exclusive breastfeeding) or changes in the food environment towards a reduction in the availability of energy‐dense nutrient‐poor foods, among others (Kennedy‐Wood et al., 2020). Notwithstanding the attention that double‐duty actions have generated and the potential effect that these may have in addressing multiple forms of malnutrition, the number of studies examining the impact of double‐duty actions is very low (Menon & Peñalvo, 2020). Identifying specific interventions that simultaneously address maternal overweight/obesity and childhood anaemia is further hindered by the inability to determine the country‐specific cause or causes of anaemia with the available data. Hence, to be able to design appropriate interventions to tackle the double burden of maternal overweight/obesity and childhood anaemia, we first need a more comprehensive assessment of what is driving anaemia in different contexts (SPRING, 2017). Failure to do so, programmes will have a limited impact in reducing anaemia and the double burden of overweight/obesity and anaemia at the three levels.

## 5 CONCLUSION

There is a high burden of the intra‐household double burden of overweight/obesity and anaemia in the 49 LMICs included in our study, primarily driven by households with overweight/obesity among mothers and anaemia among children. South Africa bears the highest burden of any intra‐household dual burden with almost one in three households affected. Large inequalities exist in the distribution of mothers with overweight/obesity and children with anaemia, with the highest prevalence observed in the richest wealth quintile, highest maternal education level and urban areas. Double‐duty approaches that target maternal overweight/obesity and childhood anaemia concurrently might help accelerate action towards reducing malnutrition in all its forms; nevertheless, understanding what causes anaemia in each LMIC is first needed to design effective interventions.

## CONFLICT OF INTERESTS

The authors declare that there are no conflict of interests.

## AUTHOR CONTRIBUTIONS

AI, PG, and RC conceptualised the study and formulated the research questions. A accessed the data, did the statistical analysis, interpreted the results and wrote the first draft of the report. All authors critically reviewed the manuscript and approved the final version.

## Supporting information

Supporting information.

## Data Availability

The authors used data from the Demographic and Health Surveys, which are publicly available and can be accessed from https://dhsprogram.com.
