# Supplementary material for: Intra‐household double burden of overweight/obesity and anaemia: Evidence from 49 low‐and middle‐income countries
Source: Matern Child Nutr. 2021 Dec 22;18(2):e13298. doi: 10.1111/mcn.13298 (PMC8932724; doi:10.1111/mcn.13298)
Supplement: Supplementary file 1 — Supporting information. [file MCN-18-e13298-s001.docx]

**Supplemental Table 1.** Sociodemographic characteristics of households (mother-child pairs) included in the study.

| **Country and survey year** | **Sample size, n** | **Urban (%)** | **Q1 (%)** | **Q5 (%)** | **E1 (%)** | **E4 (%)** |
| --- | --- | --- | --- | --- | --- | --- |
| **African region** |  |  |  |  |  |  |
| Benin (2017-18) | 3,507 | 39.3 [36.5, 42.2] | 18.3 [16.0, 20.9] | 19.6 [17.5, 21.9] | 65.0 [62.8, 67.2] | 1.4 [1.0, 2.0] |
| Burkina Faso (2010) | 4,049 | 19.7 [18.2, 21.3] | 20.1 [18.4, 22.0] | 17.6 [16.0, 19.4] | 81.5 [79.9, 82.9] | 0.4 [0.3, 0.7] |
| Burundi (2016-17) | 3,399 | 9.4 [7.7, 11.4] | 22.8 [21.1, 24.5] | 16.0 [14.1, 18.2] | 46.0 [43.8, 48.2] | 0.8 [0.5, 1.3] |
| Cameroon (2011) | 2,919 | 46.2 [43.3, 49.1] | 20.2 [18.0, 22.6] | 18.7 [16.7, 20.8] | 24.8 [22.1, 27.6] | 3.5 [2.8, 4.5] |
| Congo (2011-12) | 2,704 | 63.0 [59.6, 66.3] | 20.7 [18.4, 23.1] | 17.1 [14.2, 20.5] | 5.9 [4.7, 7.3] | 4.0 [2.9, 5.6] |
| Cote d'Ivoire (2011-12) | 1,971 | 38.6 [34.7, 42.6] | 23.7 [19.9, 28.1] | 15.4 [12.8, 18.4] | 63.4 [59.6, 67.0] | 0.6 [0.3, 1.3] |
| DRC (2013-14) | 4,096 | 30.4 [26.8, 34.2] | 22.3 [19.9, 24.9] | 16.4 [14.0, 19.2] | 19.5 [17.3, 21.9] | 1.5 [1.1, 2.2] |
| Eswatini (2006-07) | 1,495 | 19.1 [16.5, 22.0] | 20.7 [18.1, 23.7] | 17.6 [15.0, 20.6] | 8.3 [6.7, 10.1] | 6.5 [5.2, 8.2] |
| Ethiopia (2016) | 5,281 | 13.3 [11.7, 15.1] | 20.9 [17.8, 24.4] | 17.2 [15.2, 19.4] | 63.5 [60.8, 66.1] | 2.9 [2.1, 3.9] |
| Gabon (2012) | 2,036 | 85.9 [83.6, 88.0] | 18.8 [16.4, 21.6] | 18.5 [14.5, 23.3] | 6.6 [4.7, 9.1] | 7.7 [5.6, 10.5] |
| Gambia (2013) | 1,868 | 48.3 [43.9, 52.7] | 19.9 [17.3, 22.8] | 18.8 [15.6, 22.5] | 58.4 [54.7, 61.9] | 3.3 [1.9, 5.6] |
| Ghana (2014) | 1,727 | 47.6 [43.9, 51.3] | 21.7 [18.9, 24.8] | 18.3 [15.4, 21.6] | 27.0 [23.8, 30.6] | 3.3 [2.4, 4.7] |
| Guinea (2018) | 2,103 | 29.6 [27.1, 32.2] | 23.0 [20.4, 25.8] | 16.3 [14.3, 18.6] | 75.5 [73.1, 77.8] | 2.2 [1.6, 3.0] |
| Lesotho (2014) | 1,039 | 25.8 [21.6, 30.6] | 20.1 [17.1, 23.5] | 16.9 [13.5, 20.9] | 0.9 [0.5, 1.6] | 6.7 [5.1, 8.8] |
| Malawi (2015-16) | 3,672 | 14.4 [13.3, 15.6] | 23.3 [21.7, 25.0] | 17.4 [15.8, 19.0] | 12.8 [11.5, 14.3] | 2.2 [1.3, 3.4] |
| Mali (2018) | 2,374 | 22.0 [19.2, 25.2] | 19.4 [16.7, 22.4] | 18.5 [15.8, 21.5] | 71.7 [68.9, 74.4] | 1.6 [1.1, 2.3] |
| Mozambique (2011) | 5,671 | 29.5 [27.5, 31.6] | 21.5 [19.5, 23.7] | 18.0 [16.2, 19.9] | 35.8 [33.5, 38.1] | 0.7 [0.5, 1.0] |
| Namibia (2013) | 1,313 | 44.5 [41.6, 47.4] | 22.4 [19.5, 25.6] | 12.3 [9.8, 15.2] | 6.3 [5.1, 7.9] | 5.3 [3.8, 7.2] |
| Niger (2012) | 2,553 | 15.8 [14.2, 17.5] | 18.5 [16.4, 20.8] | 19.7 [17.4, 22.3] | 85.0 [83.3, 86.5] | 0.4 [0.3, 0.7] |
| Nigeria (2018) | 6,244 | 44.8 [42.8, 46.8] | 18.1 [16.6, 19.8] | 20.8 [19.0, 22.7] | 36.8 [34.9, 38.8] | 10.1 [8.9, 11.4] |
| Rwanda (2014-15) | 2,400 | 16.1 [14.7, 17.5] | 24.4 [22.5, 26.4] | 16.6 [15.1, 18.2] | 14.6 [13.0, 16.2] | 1.9 [1.4, 2.6] |
| STP (2008-09) | 1,118 | 49.3 [44.3, 54.2] | 21.1 [18.0, 24.5] | 17.7 [13.7, 22.5] | 4.9 [3.6, 6.7] | 0.7 [0.2, 1.8] |
| Senegal (2010-11) | 2,208 | 42.1 [38.2, 46.0] | 20.4 [17.7, 23.3] | 17.8 [14.5, 21.7] | 69.3 [65.8, 72.5] | 1.0 [0.5, 2.2] |
| Sierra Leone (2013) | 3,098 | 26.1 [23.4, 28.9] | 21.6 [18.8, 24.7] | 15.4 [13.3, 17.7] | 68.0 [65.6, 70.3] | 1.3 [0.9, 1.9] |
| South Africa (2016) | 868 | 56.8 [52.6, 60.8] | 23.8 [19.5, 28.8] | 10.3 [7.2, 14.5] | 2.1 [1.2, 3.8] | 9.9 [6.8, 14.0] |
| Tanzania (2015-16) | 5,350 | 29.4 [27.2, 31.7] | 20.8 [18.4, 23.3] | 19.2 [17.3, 21.2] | 19.3 [17.5, 21.2] | 0.8 [0.6, 1.2] |
| Togo (2013-14) | 2,043 | 36.8 [34.0, 39.5] | 21.5 [19.1, 24.1] | 19.3 [16.9, 21.8] | 38.7 [35.1, 42.4] | 1.6 [1.0, 2.7] |
| Uganda (2016) | 2,500 | 22.8 [20.8, 24.9] | 20.1 [18.2, 22.2] | 22.0 [19.4, 24.9] | 11.2 [9.8, 12.7] | 6.9 [5.6, 8.6] |
| Zimbabwe (2015) | 3,612 | 30.3 [27.6, 33.1] | 23.5 [20.8, 26.4] | 16.8 [14.7, 19.0] | 1.3 [0.8, 2.1] | 5.1 [4.1, 6.4] |
| **Eastern Mediterranean region** | |  |  |  |  |  |
| Egypt (2014) | 9,090 | 32.4 [30.7, 34.2] | 17.0 [15.5, 18.5] | 17.6 [16.3, 19.1] | 17.9 [16.6, 19.1] | 16.0 [14.9, 17.2] |
| Yemen (2013) | 7,524 | 30.4 [28.2, 32.7] | 19.8 [17.4, 22.4] | 20.6 [18.5, 22.8] | - | - |
| **European region** |  |  |  |  |  |  |
| Albania (2017-18) | 1,940 | 55.0 [51.5, 58.3] | 23.2 [20.6, 26.0] | 17.1 [14.3, 20.3] | 1.1 [0.5, 2.3] | 25.6 [22.7, 28.8] |
| Armenia (2015-16) | 1,172 | 57.2 [54.0, 60.4] | 19.2 [16.6, 22.1] | 22.8 [18.5, 27.7] | 0.0 [0.0, 0.0] | 54.6 [51.3, 57.8] |
| Azerbaijan (2006) | 1,343 | 51.6 [47.2, 56.0] | 22.0 [18.6, 25.8] | 17.1 [14.0, 20.6] | 1.2 [0.5, 2.6] | 14.8 [12.3, 17.7] |
| Kyrgyz Republic (2012) | 2,531 | 30.2 [27.2, 33.4] | 18.7 [16.0, 21.6] | 17.2 [14.2, 20.7] | 0.0 [0.0, 0.1] | 44.4 [41.3, 47.5] |
| Moldova (2005) | 1,146 | 39.2 [36.5, 42.0] | 18.1 [15.4, 21.0] | 21.1 [18.4, 24.1] | 0.4 [0.1, 1.1] | 18.9 [16.6, 21.5] |
| Tajikistan (2017) | 3,426 | 22.7 [21.0, 24.6] | 18.7 [16.0, 21.8] | 18.2 [16.3, 20.3] | 2.5 [1.9, 3.5] | 16.0 [14.3, 18.0] |
| **Americas region** |  |  |  |  |  |  |
| Bolivia (2008) | 5,217 | 58.0 [55.8, 60.2] | 22.1 [20.0, 24.4] | 15.5 [13.9, 17.3] | 5.5 [4.6, 6.5] | 13.3 [12.0, 14.7] |
| Guatemala (2014-15) | 8,003 | 38.6 [36.7, 40.6] | 22.8 [20.9, 24.7] | 15.7 [14.4, 17.1] | 16.8 [15.6, 18.2] | 4.3 [3.7, 4.8] |
| Guyana (2009) | 1,130 | 24.7 [21.4, 28.2] | 25.3 [21.1, 30.0] | 18.3 [15.2, 21.9] | 3.0 [1.6, 5.4] | 7.1 [5.4, 9.4] |
| Haiti (2016-17) | 2,551 | 36.6 [33.7, 39.6] | 21.9 [19.2, 24.9] | 17.1 [14.6, 20.0] | 18.2 [16.0, 20.7] | 3.8 [2.9, 4.9] |
| Honduras (2011-12) | 7,272 | 46.9 [45.1, 48.7] | 22.6 [21.1, 24.2] | 15.6 [14.1, 17.2] | 4.5 [4.0, 5.1] | 5.2 [4.5, 6.0] |
| Peru (2012) | 7,043 | 67.1 [65.4, 68.8] | 21.4 [19.9, 23.0] | 13.8 [12.2, 15.6] | 2.8 [2.4, 3.4] | 24.0 [22.4, 25.7] |
| **Southeast Asian region** |  |  |  |  |  |  |
| India (2015-16) | 155,276 | 30.0 [29.5, 30.5] | 22.8 [22.5, 23.2] | 17.0 [16.6, 17.4] | 27.2 [26.8, 27.6] | 12.1 [11.8, 12.5] |
| Maldives (2016-17) | 1,937 | 30.6 [27.6, 33.8] | 21.9 [19.6, 24.4] | 13.9 [10.9, 17.4] | 1.3 [0.9, 2.0] | 20.2 [16.9, 24.0] |
| Myanmar (2015-16) | 3,161 | 23.1 [20.9, 25.4] | 26.9 [24.3, 29.7] | 15.3 [13.3, 17.6] | 15.0 [12.9, 17.5] | 8.6 [7.2, 10.2] |
| Nepal (2016) | 1,708 | 54.2 [48.9, 59.4] | 20.1 [17.1, 23.4] | 14.4 [11.6, 17.7] | 32.8 [29.7, 36.1] | 14.9 [12.8, 17.3] |
| Timor-Leste (2016) | 3,624 | 28.4 [25.6, 31.3] | 19.8 [17.5, 22.3] | 20.0 [17.6, 22.4] | 24.6 [22.8, 26.6] | 8.9 [7.6, 10.4] |
| **Western Pacific region** |  |  |  |  |  |  |
| Cambodia (2014) | 3,292 | 14.3 [12.8, 16.0] | 23.5 [20.8, 26.4] | 19.0 [17.0, 21.3] | 12.8 [11.2, 14.6] | 2.5 [1.9, 3.2] |

Values are prevalence estimates and 95% CI, unless otherwise indicated.

Q1, poorest household wealth quintile; Q5, richest household wealth quintile; E1, lowest maternal education; E4, highest maternal education; DRC, Democratic Republic of the Congo; STP, Sao Tome and Principe.

**Supplemental Table 2.** Bivariate prevalence of overweight/obesity and anaemia in households included in the study.

|  |  |  |  |  |  |
| --- | --- | --- | --- | --- | --- |
| **Country and survey year** | **Sample size, n** | **Mother with anaemia** | **At least one child with anaemia** | **Mother with OWOB** | **At least one child with OWOB** |
| **African region** |  |  |  |  |  |
| Benin (2017-18) | 3,507 | 55.9 [53.9, 57.8] | 78.5 [76.9, 80.1] | 25.3 [23.5, 27.3] | 2.3 [1.8, 3.0] |
| Burkina Faso (2010) | 4,049 | 48.2 [46.3, 50.1] | 90.6 [89.4, 91.7] | 9.5 [8.5, 10.7] | 4.9 [4.1, 5.9] |
| Burundi (2016-17) | 3,399 | 42.9 [40.8, 45.0] | 68.9 [66.9, 70.7] | 7.5 [6.4, 8.7] | 2.3 [1.8, 2.9] |
| Cameroon (2011) | 2,919 | 36.1 [34.0, 38.2] | 69.3 [67.3, 71.3] | 30.9 [28.9, 33.0] | 10.8 [9.5, 12.3] |
| Congo (2011-12) | 2,704 | 53.9 [51.0, 56.8] | 72.9 [70.1, 75.6] | 24.7 [22.1, 27.5] | 5.3 [4.0, 6.9] |
| Cote d'Ivoire (2011-12) | 1,971 | 54.1 [51.0, 57.2] | 79.5 [77.1, 81.6] | 22.9 [20.6, 25.3] | 5.5 [4.3, 7.1] |
| DRC (2013-14) | 4,096 | 37.4 [34.6, 40.2] | 68.7 [66.1, 71.2] | 15.0 [ 17.3, 17.3] | 8.3 [7.1, 9.6] |
| Eswatini (2006-07) | 1,495 | 26.9 [23.9, 30.1] | 48.4 [45.2, 51.6] | 54.6 [51.7, 57.4] | 16.6 [14.7, 18.8] |
| Ethiopia (2016) | 5,281 | 27.0 [24.7, 29.4] | 62.1 [59.5, 64.5] | 6.4 [5.5, 7.4] | 4.3 [3.4, 5.3] |
| Gabon (2012) | 2,036 | 60.7 [57.4, 63.8] | 66.2 [62.6, 69.6] | 44.4 [41.2, 47.6] | 11.7 [9.2, 14.8] |
| Gambia (2013) | 1,868 | 61.4 [58.0, 64.6] | 80.1 [76.9, 83.1] | 22.2 [19.4, 25.2] | 5.1 [3.8, 6.8] |
| Ghana (2014) | 1,727 | 41.2 [38.2, 44.3] | 69.6 [66.6, 72.4] | 40.5 [37.6, 43.5] | 4.0 [3.0, 5.3] |
| Guinea (2018) | 2,103 | 44.3 [41.7, 46.9] | 80.8 [78.6, 82.8] | 26.9 [24.5, 29.5] | 8.1 [6.6, 9.8] |
| Lesotho (2014) | 1,039 | 24.6 [21.2, 28.3] | 56.6 [52.2, 60.8] | 45.0 [41.2, 48.9] | 9.7 [7.9, 12.0] |
| Malawi (2015-16) | 3,672 | 27.7 [25.8, 29.6] | 66.6 [64.5, 68.6] | 19.3 [17.7, 21.0] | 6.3 [5.3, 7.4] |
| Mali (2018) | 2,374 | 62.1 [59.4, 64.7] | 88.1 [86.4, 89.6] | 27.1 [24.7, 29.7] | 3.0 [2.3, 3.9] |
| Mozambique (2011) | 5,671 | 52.9 [50.7, 55.0] | 74.9 [72.8, 76.9] | 13.3 [12.2, 14.5] | 14.1 [12.8, 15.5] |
| Namibia (2013) | 1,313 | 18.5 [16.0, 21.3] | 54.6 [50.9, 58.2] | 31.3 [28.4, 34.4] | 5.5 [4.2, 7.1] |
| Niger (2012) | 2,553 | 40.1 [37.4, 42.8] | 83.6 [81.6, 85.3] | 18.2 [16.5, 20.2] | 4.5 [3.5, 5.8] |
| Nigeria (2018) | 6,244 | 57.2 [55.6, 58.8] | 74.0 [72.4, 75.5] | 29.7 [28.1, 31.3] | 3.4 [2.9, 4.0] |
| Rwanda (2014-15) | 2,400 | 17.4 [15.7, 19.2] | 42.9 [40.7, 45.1] | 21.7 [20.0, 23.5] | 13.6 [12.2, 15.0] |
| STP (2008-09) | 1,118 | 40.5 [35.8, 45.4] | 70.2 [67.1, 73.2] | 35.9 [31.8, 40.2] | 17.7 [14.9, 20.9] |
| Senegal (2010-11) | 2,208 | 50.8 [47.7, 54.0] | 83.2 [80.4, 85.7] | 21.6 [19.1, 24.4] | 4.8 [3.7, 6.2] |
| Sierra Leone (2013) | 3,098 | 44.3 [41.9, 46.7] | 84.1 [82.4, 85.7] | 16.0 [14.3, 17.9] | 14.1 [12.3, 16.1] |
| South Africa (2016) | 868 | 27.3 [22.3, 32.9] | 65.4 [60.5, 70.0] | 64.8 [60.3, 69.1] | 17.4 [14.3, 21.0] |
| Tanzania (2015-16) | 5,350 | 42.1 [40.2, 44.2] | 64.4 [62.7, 66.0] | 25.8 [24.2, 27.3] | 6.3 [5.5, 7.1] |
| Togo (2013-14) | 2,043 | 43.6 [41.3, 45.9] | 76.2 [73.9, 78.3] | 27.9 [25.6, 30.3] | 3.1 [2.3, 4.1] |
| Uganda (2016) | 2,500 | 29.2 [26.9, 31.6] | 59.9 [57.4, 62.4] | 23.8 [21.7, 26.1] | 6.5 [5.5, 7.7] |
| Zimbabwe (2015) | 3,612 | 22.2 [20.6, 23.9] | 42.6 [40.3, 44.9] | 35.2 [33.2, 37.4] | 7.5 [6.5, 8.5] |
| **Eastern Mediterranean region** | |  |  |  |  |
| Egypt (2014) | 9,090 | 26.0 [24.0, 28.1] | 32.5 [30.4, 34.8] | 80.1 [78.9, 81.2] | 21.5 [20.2, 22.9] |
| Yemen (2013) | 7,524 | 71.5 [68.7, 74.2] | 89.9 [88.1, 91.4] | 26.6 [25.1, 28.2] | 4.5 [3.9, 5.2] |
| **European region** |  |  |  |  |  |
| Albania (2017-18) | 1,940 | 23.6 [20.9, 26.6] | 26.3 [23.4, 29.4] | 42.3 [39.2, 45.5] | 20.1 [17.7, 22.7] |
| Armenia (2015-16) | 1,172 | 11.6 [9.8, 13.8] | 17.4 [15.0, 20.1] | 37.0 [33.8, 40.3] | 17.6 [15.1, 20.5] |
| Azerbaijan (2006) | 1,343 | 40.0 [36.9, 4.3] | 43.7 [40.1, 47.5] | 43.8 [40.1, 47.6] | 19.7 [17.2, 22.4] |
| Kyrgyz Republic (2012) | 2,531 | 38.6 [35.8, 41.4] | 48.2 [45.4, 51.1] | 33.8 [31.7, 35.8] | 13.5 [11.8, 15.3] |
| Moldova (2005) | 1,146 | 30.5 [27.3, 33.9] | 34.2 [31.4, 37.2] | 34.6 [31.6, 37.7] | 11.4 [9.5, 13.7] |
| Tajikistan (2017) | 3,426 | 45.4 [43.1, 47.7] | 49.5 [46.8, 52.3] | 32.4 [30.5, 34.3] | 5.4 [4.5, 6.4] |
| **Americas region** |  |  |  |  |  |
| Bolivia (2008) | 5,217 | 40.2 [37.4, 43.1] | 65.9 [62.9, 68.8] | 51.7 [49.9, 53.6] | 13.5 [12.3, 14.8] |
| Guatemala (2014-15) | 8,003 | 13.2 [12.3, 14.3] | 37.4 [35.8, 39.0] | 52.2 [50.8, 53.7] | 6.2 [5.6, 6.8] |
| Guyana (2009) | 1,130 | 36.4 [33.1, 39.8] | 41.5 [37.2, 45.9] | 48.9 [45.1, 52.9] | 11.3 [9.0, 14.0] |
| Haiti (2016-17) | 2,551 | 45.7 [43.2, 48.3] | 69.8 [67.2, 72.2] | 35.2 [32.7, 37.7] | 4.4 [3.5, 5.4] |
| Honduras (2011-12) | 7,272 | 13.6 [12.6, 14.6] | 32.7 [31.2, 34.3] | 51.8 [50.2, 53.3] | 7.1 [6.4, 7.9] |
| Peru (2012) | 7,043 | 17.7 [16.5, 19.0] | 34.9 [33.3, 36.6] | 59.5 [57.9, 61.1] | 9.8 [8.8, 10.9] |
| **Southeast Asian region** |  |  |  |  |  |
| India (2015-16) | 155,276 | 56.1 [55.7, 56.5] | 62.5 [62.1, 62.9] | 16.3 [16.0, 16.6] | 3.6 [3.4, 3.7] |
| Maldives (2016-17) | 1,937 | 61.6 [58.6, 64.4] | 53.3 [50.4, 56.2] | 53.7 [50.5, 56.9] | 5.9 [4.8, 7.3] |
| Myanmar (2015-16) | 3,161 | 42.2 [40.1, 44.4] | 62.6 [60.2, 64.9] | 26.9 [24.9, 29.1] | 1.7 [1.2, 2.3] |
| Nepal (2016) | 1,708 | 43.6 [40.6, 46.7] | 56.3 [53.2, 59.3] | 17.5 [15.3, 20.0] | 1.4 [0.9, 2.0] |
| Timor-Leste (2016) | 3,624 | 23.1 [20.7, 25.7] | 48.6 [45.1, 52.2] | 12.8 [11.4, 14.3] | 8.4 [7.4, 9.6] |
| **Western Pacific region** |  |  |  |  |  |
| Cambodia (2014) | 3,292 | 43.7 [41.5, 45.8] | 60.8 [58.5, 63.1] | 17.6 [16.0, 19.3] | 3.0 [2.4, 3.9] |

Values are prevalence estimates and 95% CI, unless otherwise indicated.

DRC, Democratic Republic of the Congo; STP, Sao Tome and Principe; OWOB, overweight/obesity**.**

**Supplemental Table 3.** Prevalence of intra-household double burden of overweight/obesity and anaemia among mothers and their children under-five.

|  | **OWOB-mother &**  **Anaemic child** | | **Anaemic mother &**  **OWOB child** | | **Total DBM** | |
| --- | --- | --- | --- | --- | --- | --- |
| **Country and survey year** | **% (95% CI)^*^** | **n** | **% (95% CI)^*^** | **n** | **% (95% CI)^*^** | **n** |
| **African region** | **16.3 [14.1, 18.6]** | **75,299** | **2.9 [2.4, 3.4]** | **80,931** | **17.3 [15.1, 19.6]** | **82,379** |
| Benin 2017-18 | 18.3 [16.7, 20.0] | 3,352 | 1.1 [0.8, 1.5] | 3,469 | 18.3 [16.8, 19.9] | 3,491 |
| Burkina Faso 2010 | 7.1 [6.1., 8.1] | 3,849 | 2.4 [1.9, 3.1] | 3,960 | 8.8 [7.8, 9.9] | 4,027 |
| Burundi 2016-17 | 4.2 [3.4, 5.1] | 3,265 | 0.8 [0.6, 1.3] | 3,383 | 4.8 [3.9, 5.7] | 3,389 |
| Cameroon 2011 | 19.7 [18.0, 21.6] | 2,746 | 3.7 [3.0, 4.6] | 2,870 | 21.3 [19.5, 23.2] | 2,908 |
| Congo 2011-12 | 16.5 [14.3, 19.1] | 2,543 | 2.5 [1.8, 3.6] | 2,651 | 17.8 [15.6, 20.3] | 2,677 |
| Cote d'Ivoire 2011-12 | 16.4 [14.2, 18.7] | 1,812 | 2.7 [1.9, 3.9] | 1,870 | 17.4 [15.2, 19.8] | 1,917 |
| DRC 2013-14 | 9.0 [7.7, 10.6] | 3,880 | 3.2 [2.5, 4.2] | 4,020 | 11.5 [10.0, 13.1] | 4,085 |
| Eswatini 2006-07 | 24.2 [21.9, 26.7] | 1,345 | 4.3 [3.3, 5.6] | 1,420 | 25.4 [23.0, 27.9] | 1,448 |
| Ethiopia 2016 | 3.1 [2.5, 3.8] | 4,896 | 1.2 [0.7, 2.0] | 5,157 | 4.1 [3.3, 5.0] | 5,220 |
| Gabon 2012 | 29.3 [25.6, 33.3] | 1,885 | 7.0 [5.2, 9.4] | 1,971 | 31.6 [28.0, 35.5] | 2,020 |
| Gambia 2013 | 15.3 [13.2, 17.6] | 1,722 | 3.2 [2.2, 4.6] | 1,753 | 16.5 [14.4, 18.9] | 1,858 |
| Ghana 2014 | 24.2 [21.8, 26.9] | 1,611 | 1.6 [1.0, 2.4] | 1,697 | 23.9 [21.6, 26.5] | 1,716 |
| Guinea 2018 | 20.8 [18.7, 23.1] | 1,958 | 2.9 [2.2, 3.8] | 2,016 | 21.7 [19.5, 24.0] | 2,068 |
| Lesotho 2014 | 22.1 [19.1, 25.3] | 950 | 2.3 [1.5, 3.6] | 1,019 | 21.9 [19.0, 25.1] | 1,033 |
| Malawi 2015-16 | 10.8 [9.4, 12.2] | 3,479 | 1.8 [1.4, 2.4] | 3,609 | 11.7 [10.3, 13.2] | 3,651 |
| Mali 2018 | 22.5 [20.3, 24.8] | 2,260 | 1.6 [1.1, 2.3] | 2,316 | 22.6 [20.4, 24.9] | 2,356 |
| Mozambique 2011 | 9.2 [7.9, 10.6] | 2,750 | 7.7 [6.8, 8.6] | 5,607 | 11.5 [10.5, 12.7] | 5,650 |
| Namibia 2013 | 16.5 [13.9, 19.4] | 1,183 | 0.7 [0.4, 1.3] | 1,278 | 15.3 [12.9, 17.9] | 1,310 |
| Niger 2012 | 15.1 [13.4, 16.9] | 2,355 | 1.5 [1.0, 2.2] | 2,427 | 15.3 [13.6, 17.1] | 2,505 |
| Nigeria 2018 | 19.2 [17.9, 20.7] | 5,963 | 2.1 [1.7, 2.6] | 6,173 | 20.1 [18.7, 21.5] | 6,220 |
| Rwanda 2014-15 | 8.8 [7.7, 10.1] | 2,293 | 2.2 [1.7, 3.0] | 2,360 | 10.3 [9.1, 11.6] | 2,400 |
| STP 2008-09 | 24.2 [20.7, 28.1] | 982 | 8.5 [6.3, 11.3] | 987 | 28.6 [25.2, 32.2] | 1,051 |
| Senegal 2010-11 | 17.4 [15.2, 19.8] | 2,023 | 2.8 [2.0, 3.9] | 2,093 | 18.8 [16.8, 21.0] | 2,154 |
| Sierra Leone 2013 | 12.7 [11.2, 14.4] | 2,889 | 6.1 [5.1, 7.3] | 2,864 | 16.7 [15.1, 18.5] | 3,085 |
| South Africa 2016 | 42.2 [37.2, 47.4] | 665 | 5.3 [3.6, 7.7] | 751 | 38.7 [34.1, 43.5] | 792 |
| Tanzania 2015-16 | 14.4 [ 13.2, 15.7] | 5,107 | 2.4 [1.9, 3.1] | 5,294 | 15.7 [14.4, 17.0] | 5,339 |
| Togo 2013-14 | 20.2 [18.2, 22.5] | 1,927 | 1.4 [0.9, 2.2] | 2,017 | 20.0 [18.0, 22.3] | 2,035 |
| Uganda 2016 | 12.1 [10.5, 13.9] | 2,377 | 2.1 [1.6, 2.9] | 2,465 | 13.4 [11.8, 15.2] | 2,488 |
| Zimbabwe 2015 | 13.6 [12.2, 15.1] | 3,232 | 1.3 [0.9, 1.8] | 3,434 | 13.7 [12.4, 15.1] | 3,486 |
| **Eastern Mediterranean region** | **24.1 [22.9, 25.3]** | **5,166** | **4.1 [3.6, 4.6]** | **5,302** | **25.3 [24.1, 26.4]** | **5,530** |
| Egypt 2014 | 25.3 [23.3, 27.3] | 3,034 | 5.7 [4.8, 6.9] | 3,054 | 27.9 [26.0, 29.9] | 3,170 |
| Yemen 2013 | 20.6 [18.6, 22.8] | 2,132 | 3.5 [2.6, 4.8] | 2,248 | 21.2 [19.3, 23.3] | 2,360 |
| **European region** | **12.1 [8.4, 15.8]** | **10,566** | **4.4 [2.9, 6.0]** | **11,237** | **14.7 [10.3, 19.1]** | **11,479** |
| Albania 2017-18 | 10.6 [8.8, 12.6] | 1,583 | 5.2 [3.8, 7.0] | 1,867 | 13.1 [11.2, 15.2] | 1,906 |
| Armenia 2015-16 | 4.7 [3.4, 6.4] | 1,052 | 1.9 [1.2, 2.9] | 1,137 | 5.8 [4.4, 7.4] | 1,161 |
| Azerbaijan 2006 | 17.1 [14.5, 19.9] | 1,253 | 8.4 [6.9, 10.2] | 1,280 | 21.9 [19.5, 24.5] | 1,342 |
| Kyrgyz Republic 2012 | 14.6 [12.9, 16.5] | 2,384 | 5.9 [4.8, 7.2] | 2,484 | 18.5 [16.5, 20.7] | 2,524 |
| Moldova 2005 | 11.7 [9.7, 14.0] | 1,004 | 3.8 [2.7, 5.4] | 1,082 | 13.7 [11.7, 15.9] | 1,128 |
| Tajikistan 2017 | 14.0 [12.6, 15.5] | 3,290 | 2.4 [1.9, 3.0] | 3,387 | 15.4 [14.0, 16.9] | 3,418 |
| **Americas region** | **21.6 [18.4, 24.7]** | **25,659** | **2.0 [1.4, 2.6]** | **27,234** | **21.4 [18.0, 24.9]** | **27,476** |
| Bolivia 2008 | 33.0 [29.9, 36.1] | 1,649 | 5.3 [4.2, 6.6] | 1,746 | 33.4 [30.4, 36.4] | 1,783 |
| Guatemala 2014-15 | 17.7 [16.6, 18.8] | 7,483 | 0.9 [0.7, 1.2] | 7,928 | 17.4 [16.3, 18.5] | 7,960 |
| Guyana 2009 | 20.5 [17.0, 24.5] | 975 | 2.8 [1.6, 4.7] | 1,049 | 19.5 [16.4, 23.2] | 1,114 |
| Haiti 2016-17 | 23.3 [21.2, 25.5] | 2,393 | 2.3 [1.7, 3.1] | 2,530 | 23.7 [21.6, 25.9] | 2,543 |
| Honduras 2011-12 | 16.1 [14.9, 17.4] | 6,554 | 0.8 [0.6, 1.1] | 7,025 | 15.5 [14.4, 16.7] | 7,085 |
| Peru 2012 | 19.4 [18.1, 20.8] | 6,605 | 1.7 [1.3, 2.2] | 6,956 | 19.5 [18.3, 20.8] | 6,991 |
| **Southeast Asian region** | **12.5 [7.9, 17.2]** | **152,285** | **1.7 [0.8, 2.6]** | **158,487** | **12.6 [8.5, 16.7]** | **162,832** |
| India 2015-16 | 8.7 [8.4, 8.9] | 145,125 | 1.8 [1.7, 2.0] | 150,665 | 9.7 [9.5, 10.0] | 154,792 |
| Maldives 2016-17 | 30.9 [28.1, 33.9] | 1,629 | 3.5 [2.6, 4.8] | 1,842 | 27.9 [25.3, 30.8] | 1,901 |
| Myanmar 2015-16 | 15.5 [13.6, 17.5] | 2,745 | 0.7 [0.4, 1.1] | 3,089 | 13.6 [12.0, 15.4] | 3,140 |
| Nepal 2016 | 8.3 [6.9, 10.1] | 1,616 | 0.4 [0.2, 0.8] | 1,693 | 8.4 [6.9, 10.1] | 1,699 |
| Timor-Leste 2016 | 5.8 [4.4, 7.7] | 1,170 | 2.3 [1.5, 3.6] | 1,198 | 7.3 [5.8, 9.2] | 1,300 |
| **Western Pacific region** | - | - | - | **-** | **-** | **-** |
| Cambodia 2014 | 9.3 [8.0, 10.7] | 3,064 | 1.3 [0.9, 1.8] | 3,223 | 9.7 [8.5, 11.1] | 3,281 |
| **Overall pooled prevalence** | **16.2 [14.6, 17.9]** | **272,039** | **2.8 [2.5, 3.1]** | **286,414** | **17.2 [15.6, 18.8]** | **292,977** |

DRC, Democratic Republic of the Congo; STP, Sao Tome and Principe; OWOB, overweight/obesity; DBM, overweight/obesity and anaemia at the household level.

Values are percentages and 95% CIs; estimates account for survey design. Regional estimates are pooled prevalences and 95% CIs, calculated with the available data from countries within that region. The Western Pacific region only had one country with available data (Cambodia), thus the regional prevalence was not calculated.

**Supplemental Table 4.** Households with overweight/obesity among mothers and anaemia among children by household wealth.

|  | **Household wealth quintiles^*^** | | | | | |  |
| --- | --- | --- | --- | --- | --- | --- | --- |
| **Country and survey year** | **Poorest (Q1)** | **Poorer (Q2)** | **Middle (Q3)** | **Richer (Q4)** | **Richest (Q5)** | **Gap**^†^ | **p-value**^‡^ |
| **AFRO** | **9.3 [7.6, 10.9]** | **12.7 [10.5, 14.9]** | **15.7 [13.0, 18.4]** | **20.0 [16.7, 23.3]** | **25.5 [22.7, 28.3]** | **16.2** |  |
| Benin 2017-18 | 9.5 [7.2, 12.4] | 13.9 [11.2, 17.1] | 14.5 [11.8, 17.8] | 21.8 [18.3, 25.7] | 31.1 [27.0, 35.6] | 21.6 | 0.0000 |
| Burkina Faso 2010 | 2.0 [1.2, 3.3] | 3.5 [2.4, 5.2] | 4.4 [3.1, 6.1] | 7.3 [5.5, 9.7] | 19.9 [16.6, 23.8] | 17.9 | 0.0000 |
| Burundi 2016-17 | 1.0 [0.4, 2.3] | 2.0 [1.2, 3.3] | 3.2 [1.9, 5.5] | 5.1 [3.4, 7.6] | 11.8 [8.9, 15.4] | 10.8 | 0.0000 |
| Cameroon 2011 | 6.2 [4.3, 8.9] | 14.7 [11.9, 18.1] | 25.4 [21.7, 29.4] | 25.0 [20.9, 29.5] | 27.9 [24.0, 32.2] | 21.7 | 0.0000 |
| Congo 2011-12 | 6.9 [5.3, 8.9] | 15.3 [9.7, 23.3] | 10.9 [7.0, 16.5] | 22.1 [16.4, 28.9] | 30.4 [24.1, 37.6] | 23.5 | 0.0000 |
| Cote d'Ivoire 2011-12 | 8.7 [6.1, 12.5] | 11.0 [8.2, 14.7] | 17.2 [13.0, 22.4] | 21.8 [16.9, 27.7] | 27.8 [22.3, 34.1] | 19.1 | 0.0000 |
| DRC 2013-14 | 4.6 [2.9, 7.2] | 4.6 [3.0, 7.0] | 7.3 [5.0, 10.7] | 9.7 [6.8, 13.7] | 22.8 [19.3, 26.8] | 18.2 | 0.0000 |
| Eswatini 2006-07 | 19.3 [15.1, 24.5] | 20.5 [16.6, 25.0] | 28.3 [23.1, 34.2] | 27.7 [22.6, 33.6] | 25.9 [20.8, 31.9] | 6.6 | 0.0943 |
| Ethiopia 2016 | 2.0 [1.3, 3.3] | 1.0 [0.4, 2.4] | 1.7 [0.9, 3.2] | 2.1 [1.1, 4.0] | 10.3 [8.0, 13.2] | 8.3 | 0.0000 |
| Gabon 2012 | 20.7 [16.7, 25.5] | 26.8 [20.1, 34.8] | 35.2 [27.3, 44.0] | 35.2 [28.1, 42.9] | 27.4 [21.8, 33.9] | 6.7 | 0.0100 |
| Gambia 2013 | 13.5 [9.8, 18.2] | 13.5 [10.2, 17.6] | 15.4 [11.2, 20.7] | 18.5 [13.7, 24.5] | 15.9 [11.6, 21.5] | 2.4 | 0.4692 |
| Ghana 2014 | 10.4 [7.9, 13.6] | 19.5 [15.1, 24.7] | 25.3 [20.3, 31.1] | 33.0 [26.2, 40.5] | 35.6 [28.2, 43.7] | 25.2 | 0.0000 |
| Guinea 2018 | 8.5 [5.9, 12.0] | 16.7 [13.2, 20.9] | 19.0 [15.3, 23.4] | 30.7 [25.1, 36.9] | 36.0 [29.9, 42.6] | 27.5 | 0.0000 |
| Lesotho 2014 | 11.4 [7.5, 17.1] | 24.4 [18.4, 31.8] | 25.3 [18.8, 33.1] | 26.0 [18.5, 35.3] | 23.4 [15.4, 33.9] | 12.0 | 0.0288 |
| Malawi 2015-16 | 8.0 [5.3, 11.8] | 6.6 [5.0, 8.7] | 10.2 [7.8, 13.3] | 13.8 [10.8, 17.4] | 17.1 [13.9, 21.0] | 9.1 | 0.0000 |
| Mali 2018 | 14.6 [10.9, 19.2] | 16.2 [13.0, 20.1] | 14.6 [11.4, 18.4] | 30.5 [26.3, 35.1] | 38.3 [33.2, 43.7] | 23.7 | 0.0000 |
| Mozambique 2011 | 2.9 [1.8, 4.8] | 6.8 [4.6, 9.9] | 7.0 [4.8, 10.1] | 10.5 [7.8, 14.2] | 20.1 [16.6, 24.1] | 17.2 | 0.0000 |
| Namibia 2013 | 9.1 [5.9, 13.6] | 13.0 [9.2, 18.0] | 15.9 [11.5, 21.6] | 25.9 [18.8, 34.6] | 20.7 [14.4, 29.0] | 11.6 | 0.0002 |
| Niger 2012 | 9.3 [6.3, 13.5] | 8.9 [6.4, 12.1] | 10.9 [8.1, 14.5] | 13.7 [10.5, 17.8] | 33.0 [28.8, 37.4] | 23.7 | 0.0000 |
| Nigeria 2018 | 6.6 [5.2, 8.3] | 11.3 [9.4, 13.4] | 18.4 [15.9, 21.2] | 27.5 [23.8, 31.6] | 29.8 [26.6, 33.2] | 23.2 | 0.0000 |
| Rwanda 2014-15 | 5.5 [3.8, 8.0] | 7.7 [5.6, 10.5] | 6.8 [4.7, 9.7] | 12.0 [8.9, 16.1] | 14.5 [11.3, 18.4] | 9.0 | 0.0000 |
| STP 2008-09 | 21.1 [15.8, 27.6] | 20.5 [15.3, 26.9] | 23.3 [16.7, 31.5] | 29.3 [20.2, 40.4] | 27.9 [18.1, 40.4] | 6.8 | 0.4102 |
| Senegal 2010-11 | 9.9 [7.3, 13.3] | 12.4 [9.5, 15.9] | 19.2 [15.0, 24.2] | 21.4 [16.0, 27.9] | 24.8 [16.1, 36.1] | 14.9 | 0.0047 |
| Sierra Leone 2013 | 7.3 [5.4, 9.8] | 9.6 [7.3, 12.5] | 11.7 [9.2, 14.7] | 14.7 [11.3, 18.9] | 24.3 [19.7, 29.5] | 17.0 | 0.0000 |
| South Africa 2016 | 41.8 [32.5, 51.7] | 46.7 [37.3, 56.4] | 41.5 [32.7, 51.0] | 36.5 [25.9, 48.7] | 44.1 [27.3, 62.4] | 2.3 | 0.7741 |
| Tanzania 2015-16 | 7.5 [5.8, 9.6] | 8.0 [6.4, 10.0] | 13.1 [10.9, 15.6] | 16.4 [13.7, 19.4] | 28.3 [25.2, 31.6] | 20.8 | 0.0000 |
| Togo 2013-14 | 7.1 [5.0, 9.9] | 12.9 [9.5, 17.1] | 18.6 [14.6, 23.3] | 27.9 [22.7, 33.8] | 36.4 [30.5, 42.6] | 29.3 | 0.0000 |
| Uganda 2016 | 4.9 [3.3, 7.3] | 8.7 [6.4, 11.8] | 10.8 [8.1, 14.3] | 9.4 [6.7, 13.1] | 25.2 [20.0, 31.3] | 20.3 | 0.0000 |
| Zimbabwe 2015 | 8.8 [6.8, 11.2] | 9.1 [6.9, 11.8] | 13.1 [10.2, 16.7] | 17.9 [15.1, 21.1] | 20.6 [17.0, 24.7] | 11.8 | 0.0000 |
| **EMRO** | **12.5 [10.6, 14.5]** | **23.5 [20.9, 26.2]** | **22.2 [19.7, 24.6]** | **28.3 [25.6, 30.9]** | **24.7 [22.1, 27.3]** | **12.2** |  |
| Egypt 2014 | 29.9 [25.6, 34.5] | 27.9 [24.0, 32.2] | 23.1 [19.5, 27.1] | 25.8 [21.8, 30.2] | 20.5 [16.5, 25.1] | -9.4 | 0.0291 |
| Yemen 2013 | 5.7 [3.7, 8.8] | 13.8 [10.6, 17.8] | 20.2 [15.9, 25.4] | 30.9 [25.8, 36.5] | 32.9 [27.8, 38.5] | 27.2 | 0.0000 |
| **EURO** | **13.2 [9.5, 16.9]** | **13.2 [7.9, 18.4]** | **12.2 [8.9, 15.5]** | **11.7 [7.8, 15.6]** | **9.4 [5.6, 13.2]** | **-3.8** |  |
| Albania 2017-18 | 12.2 [9.2, 15.9] | 12.1 [8.8, 16.4] | 13.2 [8.8, 19.3] | 7.3 [4.5, 11.6] | 6.4 [2.6, 15.2] | -5.8 | 0.1756 |
| Armenia 2015-16 | 5.5 [3.1, 9.5] | 3.7 [1.6, 8.2] | 6.3 [3.4, 11.4] | 4.1 [1.9, 8.5] | 4.1 [1.6, 10.0] | -1.4 | 0.7801 |
| Azerbaijan 2006 | 15.5 [11.7, 20.1] | 17.8 [14.3, 21.9] | 18.9 [14.7, 23.8] | 17.2 [13.1, 22.1] | 16.0 [12.9, 19.7] | 0.5 | 0.9128 |
| Kyrgyz Republic 2012 | 16.5 [13.0, 20.6] | 17.5 [14.2, 21.3] | 10.4 [7.6, 14.0] | 15.1 [11.8, 19.1] | 13.8 [9.0, 20.5] | -2.7 | 0.1171 |
| Moldova 2005 | 13.3 [9.1, 18.9] | 13.7 [9.8, 18.8] | 11.7 [8.9, 15.2] | 13.8 [11.7, 16.2] | 6.4 [3.8, 10.5] | -6.9 | 0.1460 |
| Tajikistan 2017 | 15.8 [12.4, 19.9] | 13.4 [10.5, 16.9] | 14.3 [11.6, 17.5] | 14.0 [11.1, 17.4] | 12.3 [10.1, 14.9] | -3.5 | 0.6331 |
| **PAHO** | **18.3 [14.8, 21.8]** | **20.6 [16.6, 24.5]** | **24.4 [20.7, 28.1]** | **23.9 [18.7, 29.0]** | **19.8 [14.8, 24.7]** | **1.5** |  |
| Bolivia 2008 | 26.5 [22.0, 31.5] | 34.0 [28.5, 39.9] | 42.1 [34.9, 49.7] | 34.5 [28.6, 40.8] | 25.6 [19.1, 33.4] | -0.9 | 0.0015 |
| Guatemala 2014-15 | 16.9 [14.8, 19.2] | 17.1 [15.1, 19.2] | 20.0 [17.4, 22.7] | 18.1 [16.0, 20.4] | 16.1 [13.6, 18.9] | -0.8 | 0.1886 |
| Guyana 2009 | 21.8 [14.5, 31.3] | 20.2 [13.6, 28.9] | 19.6 [13.7, 27.3] | 21.7 [14.8, 30.6] | 18.5 [11.6, 28.2] | -3.3 | 0.9686 |
| Haiti 2016-17 | 12.7 [9.5, 16.8] | 14.0 [11.0, 17.7] | 24.6 [20.5, 29.4] | 35.5 [30.6, 40.7] | 31.8 [26.3, 37.7] | 19.1 | 0.0000 |
| Honduras 2011-12 | 13.8 [12.2, 15.7] | 15.7 [13.7, 17.8] | 19.6 [17.0, 22.5] | 17.2 [14.3, 20.5] | 14.1 [11.0, 18.0] | 0.3 | 0.0148 |
| Peru 2012 | 21.0 [18.6, 23.6] | 21.8 [19.6, 24.1] | 21.8 [18.9, 25.1] | 18.3 [15.0, 22.1] | 10.2 [7.4, 14.0] | -10.8 | 0.0000 |
| **SEARO** | **8.6 [3.7, 13.6]** | **10.6 [5.1, 16.0]** | **12.8 [7.3, 18.4]** | **14.3 [9.7, 18.9]** | **16.8 [12.9, 20.8]** | **8.2** |  |
| India 2015-16 | 2.6 [2.4, 2.8] | 5.3 [5.0, 5.7] | 9.0 [8.5, 9.6] | 12.8 [12.2, 13.5] | 16.2 [15.5, 17.1] | 13.6 | 0.0000 |
| Maldives 2016-17 | 26.8 [22.6, 31.4] | 29.7 [25.4, 34.5] | 27.4 [22.5, 33.0] | 44.0 [35.1, 53.3] | 30.8 [18.5, 46.6] | 4.0 | 0.0169 |
| Myanmar 2015-16 | 9.4 [6.7, 12.9] | 14.0 [11.0, 17.6] | 18.7 [14.8, 23.3] | 18.0 [13.8, 23.0] | 23.5 [17.9, 30.2] | 14.1 | 0.0000 |
| Nepal 2016 | 6.4 [3.5, 11.6] | 3.8 [2.3, 6.4] | 5.1 [3.1, 8.1] | 11.1 [7.8, 15.5] | 18.9 [13.6, 25.5] | 12.5 | 0.0000 |
| Timor-Leste 2016 | 2.9 [1.3, 6.5] | 4.3 [2.0, 8.8] | 6.2 [3.5, 10.5] | 6.0 [3.2, 11.1] | 10.0 [6.3, 15.5] | 7.1 | 0.0576 |
| **WPRO** | **-** | **-** | **-** | **-** | **-** | **-** |  |
| Cambodia 2014 | 7.0 [5.1, 9.6] | 10.9 [8.2, 14.3] | 10.2 [7.0, 14.6] | 9.1 [6.7, 12.2] | 9.8 [7.0, 13.5] | 2.8 | 0.3414 |
| **Overall pooled prevalence** | **11.2 [9.6, 12.7]** | **13.9 [12.1, 15.7]** | **16.1 [14.3, 18.0]** | **19.0 [17.0, 21.0]** | **21.7 [19.7, 23.7]** | **10.5** |  |

AFRO, African region; DRC, Democratic Republic of the Congo; STP, Sao Tome and Principe; EMRO, Eastern Mediterranean region; EURO, European region; PAHO, Americas region; SEARO, Southeast Asian region; WPRO, Western Pacific region.

*Values are percentages and 95% CIs; estimates account for survey design. Regional estimates are pooled prevalences and 95% CIs, calculated with the available data from countries within that region. The WPRO region only had one country with available data (Cambodia), thus the regional prevalence was not calculated.

† Gaps are expressed in percentage points and indicate the difference between the highest and lowest household wealth quintile (Q5-Q1).

^‡^ p-values <0.05 indicate significant differences in the distribution of concurrent overweight/obesity and anaemia by household wealth quintiles.

**Supplemental Table 5.** Households with overweight/obesity among mothers and anaemia among children by maternal education level.

|  | **Maternal education level^*^** | | | | | |
| --- | --- | --- | --- | --- | --- | --- |
| **Country and survey year** | **None (E1)** | **Primary (E2)** | **Secondary (E3)** | **Higher (E4)** | **Gap**^†^ | **p-value**^‡^ |
| **AFRO** | **12.1 [9.4, 14.8]** | **14.6 [12.2, 17.0]** | **18.9 [16.4, 21.4]** | **22.6 [18.4, 26.9]** | **10.5** |  |
| Benin 2017-18 | 16.4 [14.5, 18.5] | 20.5 [17.3, 24.2] | 21.6 [18.0, 25.6] | 41.9 [29.1, 55.9] | 25.5 | 0.0000 |
| Burkina Faso 2010^¶^ | 5.0 [4.2, 5.9] | 14.7 [11.4, 18.6] | 19.8 [14.4, 26.6] | - | - | - |
| Burundi 2016-17 | 2.7 [1.9, 3.8] | 4.7 [3.2, 6.7] | 8.4 [5.8, 12.0] | 8.4 [3.0, 21.3] | 5.7 | 0.0006 |
| Cameroon 2011 | 8.3 [6.2, 11.2] | 20.8 [18.3, 23.5] | 26.7 [23.6, 30.1] | 28.3 [20.1, 38.3] | 20.0 | 0.0000 |
| Congo 2011-12 | 13.7 [8.0, 22.7] | 12.4 [9.1, 16.6] | 17.9 [15.1, 21.0] | 33.8 [18.3, 53.9] | 20.1 | 0.0038 |
| Cote d'Ivoire 2011-12^¶^ | 14.6 [12.0, 17.7] | 18.9 [14.8, 23.7] | 20.2 [14.0, 28.4] | - | - | - |
| DRC 2013-14 | 4.6 [2.9, 7.2] | 8.4 [6.5, 10.6] | 11.6 [9.6, 14.1] | 22.3 [11.5, 38.8] | 17.7 | 0.0000 |
| Eswatini 2006-07 | 17.3 [13.6, 21.8] | 25.8 [21.8, 30.2] | 24.9 [21.9, 28.1] | 18.3 [10.9, 28.9] | 1.0 | 0.2049 |
| Ethiopia 2016 | 2.0 [1.5, 2.8] | 3.8 [2.6, 5.4] | 7.1 [4.4, 11.4] | 15.0 [8.6, 24.8] | 13.0 | 0.0000 |
| Gabon 2012 | 30.8 [18.2, 47.3] | 32.1 [27.1, 37.6] | 27.9 [23.6, 32.6] | 29.5 [18.5, 43.4] | -1.3 | 0.6963 |
| Gambia 2013 | 16.4 [13.7, 19.5] | 13.4 [8.5, 20.5] | 15.2 [11.1, 20.5] | 1.0 [0.1, 7.5] | -15.4 | 0.1096 |
| Ghana 2014 | 15.8 [12.8, 19.4] | 25.1 [20.2, 30.8] | 27.4 [23.1, 32.1] | 43.8 [22.0, 68.2] | 28.0 | 0.0014 |
| Guinea 2018 | 19.0 [16.8, 21.4] | 21.0 [15.8, 27.4] | 32.2 [25.1, 40.3] | 27.5 [13.9, 47.2] | 8.5 | 0.0008 |
| Lesotho 2014^¶^ | - | 19.8 [15.6, 24.7] | 22.7 [17.9, 28.2] | 33.4 [22.3, 46.8] | - | - |
| Malawi 2015-16 | 15.5 [11.8, 20.1] | 9.2 [7.8, 10.9] | 12.4 [9.7, 15.8] | 12.3 [5.2, 26.6] | -3.2 | 0.0072 |
| Mali 2018 | 19.9 [17.4, 22.6] | 24.8 [19.4, 31.1] | 33.0 [27.8, 38.5] | 33.7 [17.7, 54.4] | 13.8 | 0.0001 |
| Mozambique 2011 | 6.2 [4.8, 8.0] | 10.1 [8.3, 12.4] | 12.9 [9.6, 17.1] | 14.0 [6.0, 29.4] | 7.8 | 0.0006 |
| Namibia 2013 | 14.1 [8.5, 22.5] | 12.9 [9.5, 17.3] | 18.0 [14.6, 21.9] | 15.0 [7.9, 26.7] | 0.9 | 0.2814 |
| Niger 2012^¶^ | 13.6 [11.9, 15.6] | 19.9 [15.4, 25.3] | 28.2 [21.0, 36.7] | - | - | - |
| Nigeria 2018 | 10.7 [9.2, 12.4] | 20.5 [16.3, 25.4] | 23.8 [21.8, 25.9] | 32.0 [27.4, 36.9] | 21.3 | 0.0000 |
| Rwanda 2014-15 | 5.7 [3.7, 8.7] | 8.7 [7.3, 10.3] | 13.7 [9.6, 19.1] | 11.3 [5.7, 21.2] | 5.6 | 0.0131 |
| STP 2008-09^¶^ | 24.3 [11.1, 45.2] | 23.7 [19.6, 28.2] | 26.3 [18.3, 36.3] | - | - | - |
| Senegal 2010-11^¶^ | 15.1 [12.8, 17.8] | 22.5 [17.1, 29.0] | 22.0 [14.7, 31.6] | - | - | - |
| Sierra Leone 2013 | 12.5 [10.7, 14.6] | 10.4 [7.6, 14.3] | 15.2 [11.5, 20.0] | 15.8 [8.7, 27.2] | 3.3 | 0.3007 |
| South Africa 2016^¶^ | - | 37.9 [28.4, 48.5] | 44.0 [38.3, 49.7] | 39.5 [24.9, 56.3] | - | - |
| Tanzania 2015-16 | 10.2 [8.3, 12.5] | 14.2 [12.7, 15.9] | 19.6 [16.8, 22.8] | 34.2 [20.1, 51.8] | 24.0 | 0.0000 |
| Togo 2013-14^¶^ | 14.6 [12.2, 17.3] | 23.5 [19.8, 27.7] | 23.6 [18.5, 29.5] | - | - | - |
| Uganda 2016 | 11.0 [7.5, 16.0] | 9.4 [7.6, 11.5] | 18.5 [14.8, 22.9] | 16.8 [10.5, 25.7] | 5.8 | 0.0001 |
| Zimbabwe 2015 | 9.6 [4.3, 20.0] | 9.4 [7.5, 11.6] | 15.3 [13.4, 17.4] | 19.9 [14.2, 27.1] | 10.3 | 0.0001 |
| **EMRO** | **-** | **-** | **-** | **-** | **-** |  |
| Egypt 2014 | 23.4 [19.5, 27.9] | 27.9 [21.8, 35.0] | 25.9 [23.4, 28.5] | 23.3 [19.1, 28.1] | -0.1 | 0.5061 |
| Yemen 2013^§^ | - | - | - | - | - | - |
| **EURO** | **-** | **-** | **-** | **-** | **-** |  |
| Albania 2017-18^¶^ | - | 13.3 [10.8, 16.3] | 6.3 [4.4, 9.0] | 9.5 [5.9, 14.8] | - | - |
| Armenia 2015-16^¶^ | - | 3.7 [1.0, 12.6] | 5.8 [3.7, 9.0] | 3.9 [2.4, 6.4] | - | - |
| Azerbaijan 2006^¶^ | - | - | 16.8 [14.1, 19.8] | 17.2 [11.9, 24.3] | - | - |
| Kyrgyz Republic 2012^¶^ | - | - | 15.4 [13.1, 18.0] | 13.5 [11.1, 16.3] | - | - |
| Moldova 2005^¶^ | - | - | 11.7 [9.7, 14.1] | 11.1 [9.4, 13.1] | - | - |
| Tajikistan 2017 | 17.0 [9.5, 28.5] | 18.7 [13.1, 25.8] | 13.8 [12.2, 15.7] | 12.5 [9.7, 16.0] | -4.5 | 0.2965 |
| **PAHO** | **20.8 [15.9, 25.7]** | **21.9 [18.6, 25.3]** | **21.8 [17.5, 26.2]** | **19.8 [16.2, 23.4]** | **-1.0** |  |
| Bolivia 2008 | 38 6 [25.6, 53.5] | 32.3 [28.3, 36.5] | 37.1 [31.7, 42.8] | 24.4 [18.5, 31.4] | -14.2 | 0.0372 |
| Guatemala 2014-15 | 19.2 [16.6, 22.2] | 18.4 [17.0, 19.9] | 15.7 [13.8, 17.8] | 15.8 [12.0, 20.5] | -3.4 | 0.0784 |
| Guyana 2009 | 22.3 [8.8, 45.8] | 22.6 [14.8, 32.8] | 20.1 [16.4, 24.3] | 17.5 [7.0, 37.3] | -4.8 | 0.8714 |
| Haiti 2016-17 | 14.1 [10.8, 18.2] | 24.6 [21.5, 27.9] | 25.7 [22.4, 29.3] | 30.9 [19.3, 45.4] | 16.8 | 0.0004 |
| Honduras 2011-12 | 19.7 [15.4, 24.8] | 17.6 [16.2, 19.2] | 12.6 [10.8, 14.7] | 17.5 [12.1, 24.7] | -2.2 | 0.0007 |
| Peru 2012 | 26.8 [20.6, 34.2] | 21.7 [19.5, 24.1] | 20.4 [18.5, 22.6] | 13.8 [11.6, 16.3] | -13.0 | 0.0000 |
| **SEARO** | **6.4 [4.0, 8.8]** | **12.7 [6.8, 18.7]** | **12.9 [7.7, 18.1]** | **14.9 [10.8, 18.9]** | **8.5** |  |
| India 2015-16 | 5.1 [4.8, 5.4] | 6.7 [6.2, 7.3] | 9.9 [9.6, 10.3] | 14.5 [13.6, 15.4] | 9.4 | 0.0000 |
| Maldives 2016-17 | 34.5 [12.5, 65.9] | 34.9 [28.7, 41.7] | 31.2 [27.5, 35.2] | 25.3 [19.3, 32.4] | -9.2 | 0.2277 |
| Myanmar 2015-16 | 10.4 [7.0, 15.1] | 16.1 [13.4, 19.1] | 14.8 [11.9, 18.4] | 23.5 [16.9, 31.6] | 13.1 | 0.0162 |
| Nepal 2016 | 7.4 [5.3, 10.3] | 7.6 [4.7, 12.0] | 9.6 [7.0, 13.1] | 8.6 [5.3, 13.5] | 1.2 | 0.6793 |
| Timor-Leste 2016 | 3.9 [1.8, 8.0] | 4.2 [2.0, 8.3] | 6.8 [4.7, 9.7] | 10.0 [4.4, 20.9] | 6.1 | 0.1715 |
| **WPRO** | **-** | **-** | **-** | **-** | **-** |  |
| Cambodia 2014 | 9.3 [6.6, 13.1] | 10.0 [8.2, 12.2] | 7.5 [5.6, 9.8] | 15.0 [8.0, 26.3] | 5.7 | 0.1701 |
| **Overall pooled prevalence** | **13.1 [11.3, 15.0]** | **15.9 [13.8, 18.0]** | **18.3 [16.0, 20.6]** | **19.4 [17.3, 21.6]** | **6.3** |  |

AFRO, African region; DRC, Democratic Republic of the Congo; STP, Sao Tome and Principe; EMRO, Eastern Mediterranean region; EURO, European region; PAHO, Americas region; SEARO, Southeast Asian region; WPRO, Western Pacific region.

*Values are percentages and 95% CIs; estimates account for survey design. Regional estimates are pooled prevalences and 95% CIs, calculated with the available data from countries within that region. The WPRO region only had one country with available data (Cambodia), thus the regional prevalence was not calculated.

† Gaps are expressed in percentage points and indicate the difference between the highest and lowest education level (E4-E1).

^‡^ p-values <0.05 indicate significant differences in the distribution of concurrent overweight/obesity and anaemia by education level.

^§^ Yemen has missing data on education level, and thus, the stratified estimates could not be calculated.

^¶^ Estimates for certain categories are missing due to sample size <25.

**Supplemental Table 6.** Households with overweight/obesity among mothers and anaemia among children by area of residence.

|  | **Area of residence^*^** | | | |
| --- | --- | --- | --- | --- |
| **Country and survey year** | **Urban** | **Rural** | **Gap**^†^ | **p-value**^‡^ |
| **AFRO** | **22.9 [20.6, 25.2]** | **13.3 [11.2, 15.3]** | **9.6** |  |
| Benin 2017-18 | 25.0 [22.2, 28.1] | 14.0 [12.3, 15.9] | 11.0 | 0.0000 |
| Burkina Faso 2010 | 19.4 [16.1, 23.2] | 4.1 [3.4, 5.0] | 15.3 | 0.0000 |
| Burundi 2016-17 | 15.1 [11.0, 20.5] | 3.0 [2.3, 3.9] | 12.1 | 0.0000 |
| Cameroon 2011 | 25.8 [23.1, 28.8] | 14.4 [12.4, 16.7] | 11.4 | 0.0000 |
| Congo 2011-12 | 21.8 [18.3, 25.7] | 7.8 [6.5, 9.3] | 14.0 | 0.0000 |
| Cote d'Ivoire 2011-12 | 24.4 [20.6, 28.6] | 11.2 [8.9, 14.1] | 13.2 | 0.0000 |
| DRC 2013-14 | 17.4 [14.6, 20.7] | 5.4 [4.2, 6.9] | 12.0 | 0.0000 |
| Eswatini 2006-07 | 24.2 [19.1, 30.2] | 24.2 [21.6, 27.0] | 0.0 | 0.9854 |
| Ethiopia 2016 | 12.4 [9.4, 16.1] | 1.8 [1.3, 2.4] | 10.6 | 0.0000 |
| Gabon 2012 | 30.5 [26.3, 35.1] | 21.5 [18.5, 24.9] | 9.0 | 0.0010 |
| Gambia 2013 | 15.1 [11.8, 19.0] | 15.5 [13.0, 18.4] | -0.4 | 0.8353 |
| Ghana 2014 | 30.9 [27.0, 35.1] | 18.3 [15.6, 21.4] | 12.6 | 0.0000 |
| Guinea 2018 | 33.8 [28.8, 39.1] | 15.7 [13.6, 18.0] | 18.1 | 0.0000 |
| Lesotho 2014 | 21.4 [15.3, 29.1] | 22.3 [19.0, 25.9] | -0.9 | 0.8169 |
| Malawi 2015-16 | 18.1 [13.8, 23.5] | 9.5 [8.2, 11.1] | 8.6 | 0.0001 |
| Mali 2018 | 38.0 [33.3, 42.9] | 18.1 [15.9, 20.7] | 19.9 | 0.0000 |
| Mozambique 2011 | 12.1 [9.9, 14.8] | 7.9 [6.5, 9.7] | 4.2 | 0.0032 |
| Namibia 2013 | 21.6 [17.0, 27.1] | 12.3 [9.9, 15.2] | 9.3 | 0.0005 |
| Niger 2012 | 36.4 [31.9, 41.1] | 11.2 [9.5, 13.1] | 25.2 | 0.0000 |
| Nigeria 2018 | 25.2 [22.9, 27.7] | 14.4 [13.0, 15.8] | 10.8 | 0.0000 |
| Rwanda 2014-15 | 14.5 [11.6, 18.0] | 7.7 [6.5, 9.2] | 6.8 | 0.0000 |
| STP 2008-09 | 27.4 [22.2, 33.2] | 20.3 [15.9, 25.6] | 7.1 | 0.0555 |
| Senegal 2010-11 | 25.2 [21.2, 29.6] | 11.7 [9.8, 13.9] | 13.5 | 0.0000 |
| Sierra Leone 2013 | 21.2 [17.5, 25.5] | 9.8 [8.3, 11.5] | 11.4 | 0.0000 |
| South Africa 2016 | 43.3 [35.7, 51.2] | 40.8 [35.0, 46.9] | 2.5 | 0.6132 |
| Tanzania 2015-16 | 23.1 [20.4, 26.1] | 10.8 [9.7, 12.1] | 12.3 | 0.0000 |
| Togo 2013-14 | 31.1 [27.1, 35.5] | 14.1 [12.0, 16.4] | 17.0 | 0.0000 |
| Uganda 2016 | 20.1 [15.5, 25.6] | 9.8 [8.3, 11.5] | 10.3 | 0.0000 |
| Zimbabwe 2015 | 19.2 [16.5, 22.2] | 11.2 [9.6, 12.9] | 8.0 | 0.0000 |
| **EMRO** | **26.3 [24.3, 28.3]** | **22.1 [20.7, 23.5]** | **4.2** |  |
| Egypt 2014 | 23.1 [19.9, 26.6] | 26.3 [23.9, 28.9] | -3.2 | 0.1296 |
| Yemen 2013 | 32.7 [28.2, 37.5] | 15.1 [13.2, 17.3] | 17.6 | 0.0000 |
| **EURO** | **11.0 [7.3, 14.8]** | **12.9 [9.3, 16.4]** | **-1.9** |  |
| Albania 2017-18 | 8.8 [6.4, 12.1] | 12.5 [10.3, 15.2] | -3.7 | 0.0612 |
| Armenia 2015-16 | 4.9 [3.1, 7.6] | 4.5 [2.9, 7.0] | 0.4 | 0.7923 |
| Azerbaijan 2006 | 19.5 [15.8, 23.7] | 14.5 [11.2, 18.5] | 5.0 | 0.0681 |
| Kyrgyz Republic 2012 | 14.8 [11.1, 19.5] | 14.5 [12.7, 16.5] | 0.3 | 0.8915 |
| Moldova 2005 | 7.7 [5.8, 10.1] | 14.0 [11.2, 17.4] | -6.3 | 0.0009 |
| Tajikistan 2017 | 12.7 [10.8, 14.9] | 14.3 [12.7, 16.2] | -1.6 | 0.2408 |
| **PAHO** | **23.3 [19.1, 27.4]** | **20.4 [17.6, 23.3]** | **2.9** |  |
| Bolivia 2008 | 34.5 [30.2, 39.2] | 30.9 [27.1, 35.1] | 3.6 | 0.2395 |
| Guatemala 2014-15 | 17.8 [16.1, 19.7] | 17.6 [16.2, 19.1] | 0.2 | 0.8468 |
| Guyana 2009 | 23.9 [16.4, 33.5] | 19.5 [15.6, 24.0] | 4.4 | 0.3332 |
| Haiti 2016-17 | 30.1 [26.5, 34.0] | 19.4 [16.9, 22.2] | 10.7 | 0.0000 |
| Honduras 2011-12 | 16.5 [14.7, 18.5] | 15.8 [14.3, 17.4] | 0.7 | 0.5488 |
| Peru 2012 | 18.2 [16.5, 19.9] | 21.9 [19.8, 24.1] | -3.7 | 0.0074 |
| **SEARO** | **15.2 [10.8, 19.5]** | **11.4 [6.0, 16.9]** | **3.8** |  |
| India 2015-16 | 14.3 [13.7, 14.9] | 6.3 [6.1, 6.5] | 8.0 | 0.0000 |
| Maldives 2016-17 | 41.9 [33.3, 50.9] | 27.0 [24.7, 29.5] | 14.9 | 0.0005 |
| Myanmar 2015-16 | 20.5 [15.8, 26.2] | 14.1 [12.2, 16.2] | 6.4 | 0.0126 |
| Nepal 2016 | 9.6 [7.4, 12.4] | 6.8 [5.0, 9.3] | 2.8 | 0.0959 |
| Timor-Leste 2016 | 9.0 [5.5, 14.3] | 4.6 [3.2, 6.5] | 4.4 | 0.0267 |
| **WPRO** | **-** | **-** | **-** |  |
| Cambodia 2014 | 10.3 [7.9, 13.4] | 9.1 [7.7, 10.7] | 1.2 | 0.4243 |
| **Overall pooled prevalence** | **20.8 [18.9, 22.7]** | **14.2 [12.7, 15.7]** | **6.6** |  |

AFRO, African region; DRC, Democratic Republic of the Congo; STP, Sao Tome and Principe; EMRO, Eastern Mediterranean region; EURO, European region; PAHO, Americas region; SEARO, Southeast Asian region; WPRO, Western Pacific region.

*Values are percentages and 95% CIs; estimates account for survey design. Regional estimates are pooled prevalences and 95% CIs, calculated with the available data from countries within that region. The WPRO region only had one country with available data (Cambodia), thus the regional prevalence was not calculated.

† Gaps are expressed in percentage points and indicate the difference between urban and rural areas (urban-rural).

^‡^ p-values <0.05 indicate significant differences in the distribution of concurrent overweight/obesity and anaemia by area of residence.

**Supplemental Table 7.** Households with anaemia among mothers and overweight/obesity among children by household wealth.

|  | **Household wealth quintiles^*^** | | | | | |  |
| --- | --- | --- | --- | --- | --- | --- | --- |
| **Country and survey year** | **Poorest (Q1)** | **Poorer (Q2)** | **Middle (Q3)** | **Richer (Q4)** | **Richest (Q5)** | **Gap**^†^ | **p-value**^‡^ |
| **AFRO** | **2.7 [2.1, 3.3]** | **2.7 [2.2, 3.3]** | **2.7 [2.1, 3.2]** | **2.5 [1.9, 3.1]** | **2.5 [2.0, 3.1]** | **-0.2** |  |
| Benin 2017-18 | 0.8 [0.3, 2.1] | 1.0 [0.5, 2.1] | 1.8 [1.0, 3.3] | 0.8 [0.4, 1.7] | 0.8 [0.4, 1.8] | 0.0 | 0.2833 |
| Burkina Faso 2010 | 3.1 [1.9, 5.0] | 2.7 [1.7, 4.3] | 2.4 [1.3, 4.4] | 2.1 [1.2, 3.4] | 1.8 [0.9, 3.5] | -1.3 | 0.6719 |
| Burundi 2016-17 | 0.8 [0.3, 1.8] | 1.1 [0.4, 2.7] | 0.9 [0.4, 2.1] | 1.0 [0.4, 2.2] | 0.4 [0.1, 1.1] | -0.4 | 0.7927 |
| Cameroon 2011 | 2.5 [1.4, 4.3] | 4.1 [2.1, 7.6] | 5.1 [3.4, 7.6] | 3.7 [2.5, 5.7] | 3.2 [1.9, 5.3] | 0.7 | 0.3878 |
| Congo 2011-12 | 1.6 [1.0, 2.5] | 3.0 [1.6, 5.4] | 2.7 [1.1, 6.7] | 2.2 [0.9, 5.5] | 3.2 [1.3, 7.3] | 1.6 | 0.7636 |
| Cote d'Ivoire 2011-12 | 2.4 [1.2, 4.8] | 2.6 [1.3, 4.9] | 2.6 [1.1, 5.6] | 2.6 [1.3, 5.1] | 3.8 [1.6, 9.1] | 1.4 | 0.8896 |
| DRC 2013-14 | 3.3 [2.1, 5.2] | 2.8 [1.6, 5.0] | 4.3 [2.8, 6.6] | 3.2 [1.8, 5.8] | 2.5 [1.4, 4.4] | -0.8 | 0.6197 |
| Eswatini 2006-07 | 2.4 [1.2, 4.5] | 5.7 [3.8, 8.5] | 3.5 [2.1, 5.8] | 5.3 [3.1, 9.0] | 4.7 [3.1, 7.2] | 2.3 | 0.3003 |
| Ethiopia 2016 | 3.1 [1.4, 6.7] | 0.5 [0.2, 1.5] | 0.8 [0.4, 1.9] | 0.6 [0.2, 1.8] | 1.0 [0.5, 2.2] | -2.1 | 0.0014 |
| Gabon 2012 | 6.9 [4.9, 9.6] | 5.3 [3.0, 9.3] | 7.7 [3.7, 15.4] | 8.0 [4.0, 15.5] | 7.2 [3.8, 13.0] | 0.3 | 0.8803 |
| Gambia 2013 | 3.4 [2.0, 5.9] | 1.5 [0.7, 3.4] | 4.8 [2.8, 7.9] | 3.7 [1.2, 10.8] | 2.8 [1.2, 6.4] | -0.6 | 0.4014 |
| Ghana 2014 | 1.2 [0.5, 2.9] | 2.0 [1.0, 4.0] | 2.4 [1.0, 6.0] | 0.6 [0.1, 2.5] | 1.7 [0.6, 5.0] | 0.5 | 0.4603 |
| Guinea 2018 | 2.8 [1.6, 4.8] | 2.2 [1.2, 4.0] | 3.0 [1.4, 6.3] | 2.3 [1.1, 4.7] | 4.7 [2.8, 8.0] | 1.9 | 0.4055 |
| Lesotho 2014 | 2.3 [0.9, 5.5] | 4.1 [2.0, 8.2] | 3.6 [1.4, 9.0] | 0.2 [0.0, 1.7] | 0.8 [0.2, 2.8] | -1.5 | 0.0454 |
| Malawi 2015-16 | 0.8 [0.4, 1.9] | 2.1 [1.3, 3.5] | 2.5 [1.5, 4.2] | 2.1 [1.2, 3.7] | 1.6 [0.7, 3.6] | 0.8 | 0.2408 |
| Mali 2018 | 1.2 [0.5, 3.1] | 2.1 [1.0, 4.4] | 1.3 [0.6, 2.9] | 2.0 [1.0, 3.9] | 1.4 [0.6, 3.4] | 0.2 | 0.7836 |
| Mozambique 2011 | 7.8 [6.0, 10.1] | 7.5 [5.7, 9.8] | 8.5 [6.3, 11.4] | 8.2 [6.4, 10.3] | 6.3 [4.8, 8.1] | -1.5 | 0.6382 |
| Namibia 2013 | 0.9 [0.2, 3.4] | 1.2 [0.4, 3.1] | 0.1 [0.0, 0.4] | 1.0 [0.4, 2.8] | 0.0 [0.0, 0.0] | -0.9 | 0.2543 |
| Niger 2012 | 2.5 [1.1, 5.4] | 0.6 [0.2, 2.0] | 1.7 [0.7, 4.2] | 0.4 [0.1, 1.3] | 2.4 [1.2, 5.0] | -0.1 | 0.0549 |
| Nigeria 2018 | 1.7 [1.0, 3.1] | 3.0 [2.1, 4.5] | 2.2 [1.5, 3.4] | 2.4 [1.4, 4.0] | 1.0 [0.5, 2.1] | -0.7 | 0.0894 |
| Rwanda 2014-15 | 3.3 [2.1, 5.2] | 3.3 [2.0, 5.2] | 1.5 [0.7, 3.5] | 1.4 [0.6, 2.9] | 1.1 [0.4, 2.7] | -2.2 | 0.0465 |
| STP 2008-09 | 8.5 [4.7, 14.8] | 8.0 [4.8, 13.1] | 6.9 [3.1, 14.6] | 6.9 [3.5, 13.4] | 12.5 [7.2, 20.9] | 4.0 | 0.5273 |
| Senegal 2010-11 | 3.2 [2.1, 4.9] | 1.8 [0.9, 3.4] | 3.0 [1.6, 5.5] | 3.8 [1.9, 7.4] | 1.9 [0.6, 6.2] | -1.3 | 0.5086 |
| Sierra Leone 2013 | 7.9 [5.8, 10.6] | 5.9 [4.0, 8.7] | 5.8 [3.9, 8.5] | 6.4 [4.4, 9.2] | 4.0 [2.4, 6.6] | -3.9 | 0.2793 |
| South Africa 2016 | 7.1 [4.2, 11.8] | 8.3 [3.9, 16.8] | 3.6 [1.6, 7.9] | 1.6 [0.5, 5.3] | 4.7 [1.2, 16.1] | -2.4 | 0.1271 |
| Tanzania 2015-16 | 2.5 [1.7, 3.5] | 2.5 [1.5, 4.3] | 2.5 [1.5, 4.3] | 2.3 [1.5, 3.5] | 2.4 [1.5, 3.8] | -0.1 | 0.9983 |
| Togo 2013-14 | 0.7 [0.2, 1.9] | 1.2 [0.4, 3.3] | 1.9 [0.8, 4.2] | 1.3 [0.4, 3.9] | 2.1 [1.0, 4.3] | 1.4 | 0.5098 |
| Uganda 2016 | 2.1 [1.1, 3.8] | 2.2 [1.2, 4.1] | 1.8 [0.9, 3.6] | 3.5 [2.0, 6.2] | 1.2 [0.5, 3.3] | -0.9 | 0.2621 |
| Zimbabwe 2015 | 0.9 [0.4, 2.1] | 1.0 [0.5, 2.3] | 0.6 [0.2, 1.4] | 1.8 [1.0, 3.1] | 2.2 [1.2, 3.8] | 1.3 | 0.1016 |
| **EMRO** | **5.2 [3.8, 6.6]** | **3.3 [2.2, 4.4]** | **3.1 [2.1, 4.1]** | **4.2 [3.1, 5.4]** | **4.2 [3.0, 5.4]** | **-1.0** |  |
| Egypt 2014 | 6.5 [4.5, 9.1] | 3.7 [ 2.4, 5.7] | 3.6 [2.2, 6.0] | 6.6 [4.3, 9.8] | 9.2 [6.8, 12.3] | 2.7 | 0.0046 |
| Yemen 2013 | 5.0 [2.8, 8.7] | 3.3 [1.6, 6.8] | 4.1 [2.3, 7.2] | 3.6 [2.2, 5.8] | 1.6 [0.8, 3.3] | -3.4 | 0.2582 |
| **EURO** | **5.1 [3.5, 6.7]** | **4.8 [3.1, 6.4]** | **3.8 [2.0, 5.6]** | **3.4 [2.2, 4.7]** | **4.5 [2.4, 6.6]** | **-0.6** |  |
| Albania 2017-18 | 5.2 [3.6, 7.7] | 5.8 [3.2, 10.1] | 6.2 [3.3, 11.5] | 3.8 [2.0, 7.1] | 4.5 [1.5, 12.2] | -0.7 | 0.8100 |
| Armenia 2015-16 | 3.0 [1.3, 6.4] | 2.3 [1.0, 5.3] | 2.3 [0.8, 6.2] | 2.2 [0.8, 5.7] | 0.0 [0.0, 0.0] | -3.0 | 0.1914 |
| Azerbaijan 2006 | 8.8 [7.0, 11.0] | 10.6 [7.4, 15.0] | 6.8 [4.7, 9.7] | 5.3 [3.6, 7.9] | 10.0 [6.5, 15.0] | 1.2 | 0.3009 |
| Kyrgyz Republic 2012 | 6.9 [4.2, 11.1] | 5.9 [4.0, 8.7] | 4.1 [2.8, 6.0] | 6.1 [3.7, 9.9] | 6.8 [4.1, 10.8] | -0.1 | 0.5883 |
| Moldova 2005 | 5.4 [3.1, 9.3] | 5.4 [2.9, 9.8] | 2.5 [1.2, 5.1] | 1.2 [0.6, 2.4] | 4.7 [2.4, 9.2] | -0.7 | 0.1143 |
| Tajikistan 2017 | 2.3 [1.3, 4.2] | 2.8 [1.7, 4.7] | 1.3 [0.7, 2.7] | 2.8 [1.7, 4.6] | 2.8 [1.7, 4.3] | 0.5 | 0.4218 |
| **PAHO** | **1.9 [1.1, 2.8]** | **1.3 [0.7, 2.0]** | **2.2 [0.6, 1.7]** | **1.9 [1.2, 2.6]** | **2.1 [1.2, 3.0]** | **0.2** |  |
| Bolivia 2008 | 8.3 [5.6, 12.2] | 5.1 [3.1, 8.4] | 2.7 [1.4, 5.3] | 5.2 [3.0, 8.9] | 4.5 [2.2, 8.8] | -3.8 | 0.0658 |
| Guatemala 2014-15 | 1.5 [1.0, 2.3] | 0.4 [0.2, 0.9] | 0.6 [0.3, 1.2] | 1.1 [0.7, 1.9] | 0.9 [0.5, 1.8] | -0.6 | 0.0152 |
| Guyana 2009 | 4.1 [1.9, 8.7] | 4.4 [1.8, 10.5] | 0.7 [0.2, 3.1] | 4.1 [1.6, 9.9] | 0.3 [0.0, 2.4] | -3.8 | 0.0348 |
| Haiti 2016-17 | 1.8 [0.9, 3.6] | 2.5 [1.4, 4.6] | 2.1 [1.1, 3.9] | 2.3 [1.2, 4.5] | 2.8 [1.3, 5.7] | 1.0 | 0.9076 |
| Honduras 2011-12 | 0.3 [0.2, 0.6] | 0.6 [0.3, 1.2] | 0.6 [0.3, 1.4] | 0.9 [0.5, 1.7] | 2.1 [1.2, 3.5] | 1.8 | 0.0003 |
| Peru 2012 | 1.2 [0.8, 2.0] | 0.8 [0.5, 1.4] | 1.5 [0.8, 2.7] | 2.2 [1.2, 3.9] | 3.6 [2.1, 6.1] | 2.4 | 0.0026 |
| **SEARO** | **1.6 [0.8, 2.4]** | **1.4 [0.7, 2.1]** | **2.2 [0.9, 3.6]** | **1.5 [0.4, 2.6]** | **1.9 [1.3, 2.6]** | **0.3** |  |
| India 2015-16 | 1.7 [1.6, 1.9] | 1.6 [1.4, 1.8] | 1.9 [1.7, 2.1] | 2.0 [1.7, 2.2] | 2.1 [1.9, 2.5] | 0.4 | 0.0135 |
| Maldives 2016-17 | 2.8 [1.6, 4.7] | 2.4 [1.4, 4.3] | 4.2 [2.8, 6.3] | 8.0 [4.0, 15.4] | 0.0 [0.0, 0.0] | -2.8 | 0.0033 |
| Myanmar 2015-16 | 0.4 [0.1, 1.0] | 0.6 [0.2, 2.1] | 0.3 [0.1, 1.8] | 0.5 [0.1, 2.0] | 1.9 [0.8, 4.4] | 1.5 | 0.0526 |
| Nepal 2016 | 0.6 [0.2, 2.2] | 1.0 [0.4, 2.6] | 0.0 [0.0, 0.0] | 0.3 [0.0, 1.9] | 0.0 [0.0, 0.0] | -0.6 | 0.3158 |
| Timor-Leste 2016 | 1.5 [0.6, 3.7] | 1.3 [0.4, 4.2] | 3.0 [1.4, 6.4] | 1.5 [0.5, 4.4] | 4.3 [1.8, 9.6] | 2.8 | 0.1910 |
| **WPRO** | **-** | **-** | **-** | **-** | **-** | **-** |  |
| Cambodia 2014 | 1.1 [0.5, 2.1] | 1.2 [0.6, 2.5] | 0.9 [0.3, 2.3] | 1.8 [0.8, 3.8] | 1.6 [0.7, 3.4] | 0.5 | 0.7018 |
| **Overall pooled prevalence** | **2.7 [2.3, 3.1]** | **2.5 [2.2, 2.9]** | **2.4 [2.1, 2.8]** | **2.4 [2.1, 2.8]** | **2.5 [2.1, 2.9]** | **-0.2** |  |

AFRO, African region; DRC, Democratic Republic of the Congo; STP, Sao Tome and Principe; EMRO, Eastern Mediterranean region; EURO, European region; PAHO, Americas region; SEARO, Southeast Asian region; WPRO, Western Pacific region.

*Values are percentages and 95% CIs; estimates account for survey design. Regional estimates are pooled prevalences and 95% CIs, calculated with the available data from countries within that region. The WPRO region only had one country with available data (Cambodia), thus the regional prevalence was not calculated.

† Gaps are expressed in percentage points and indicate the difference between the highest and lowest household wealth quintile (Q5-Q1).

^‡^ p-values <0.05 indicate significant differences in the distribution of concurrent overweight/obesity and anaemia by household wealth quintiles.

**Supplemental Table 8.** Households with anaemia among mothers and overweight/obesity among children by maternal education level.

|  | **Maternal education level^*^** | | | | | |
| --- | --- | --- | --- | --- | --- | --- |
| **Country and survey year** | **None (E1)** | **Primary (E2)** | **Secondary (E3)** | **Higher (E4)** | **Gap**^†^ | **p-value**^‡^ |
| **AFRO** | **2.9 [2.1, 3.6]** | **2.7 [2.1, 3.4]** | **2.7 [2.1, 3.4]** | **1.9 [1.3, 2.5]** | **-1.0** |  |
| Benin 2017-18 | 1.1 [0.7, 1.6] | 1.1 [0.5, 2.2] | 1.1 [0.5, 2.4] | 1.2 [0.2, 9.1] | 0.1 | 0.9989 |
| Burkina Faso 2010^¶^ | 2.5 [1.9, 3.3] | 1.7 [0.8, 3.6] | 2.5 [0.9, 6.5] | - | - | - |
| Burundi 2016-17 | 0.7 [0.4, 1.3] | 1.1 [0.6, 1.9] | 0.8 [0.2, 2.6] | 0.0 [0.0, 0.0] | -0.7 | 0.7123 |
| Cameroon 2011 | 2.1 [1.2, 3.5] | 4.1 [3.0, 5.6] | 4.8 [3.5, 6.5] | 1.5 [0.3, 6.3] | -0.6 | 0.0511 |
| Congo 2011-12 | 3.2 [0.8, 12.6] | 2.1 [1.3, 3.6] | 2.6 [1.6, 4.2] | 2.6 [0.5, 13.2] | -0.6 | 0.9226 |
| Cote d'Ivoire 2011-12^¶^ | 3.3 [2.2, 4.9] | 1.8 [0.9, 3.7] | 1.4 [0.2, 8.3] | - | - | - |
| DRC 2013-14 | 3.0 [1.8, 5.1] | 2.8 [1.9, 4.0] | 4.0 [2.6, 6.0] | 1.4 [0.2, 10.1] | -1.6 | 0.3929 |
| Eswatini 2006-07 | 5.1 [2.8, 9.1] | 3.3 [2.0, 5.5] | 4.4 [3.0, 6.2] | 8.2 [4.7, 14.1] | 3.1 | 0.2439 |
| Ethiopia 2016 | 1.0 [0.5, 1.8] | 1.5 [0.8, 2.8] | 2.8 [1.2, 6.4] | 1.0 [0.3, 3.9] | 0.0 | 0.0875 |
| Gabon 2012 | 9.3 [3.2, 24.1] | 7.5 [5.0, 10.9] | 6.9 [4.7, 9.9] | 4.9 [0.9, 21.7] | -4.4 | 0.8413 |
| Gambia 2013 | 2.9 [1.9, 4.4] | 3.1 [1.5, 6.6] | 3.7 [1.9, 7.2] | 3.8 [1.2, 11.9] | 0.9 | 0.8620 |
| Ghana 2014 | 1.9 [0.9, 4.0] | 1.6 [0.7, 3.9] | 1.3 [0.6, 2.7] | 2.7 [0.3, 19.0] | 0.8 | 0.8130 |
| Guinea 2018 | 2.7 [1.9, 3.8] | 2.4 [0.8, 6.7] | 4.9 [2.6, 9.0] | 2.8 [0.3, 19.3] | 0.1 | 0.4635 |
| Lesotho 2014^¶^ | - | 2.6 [1.4, 4.6] | 2.3 [1.1, 4.9] | 1.1 [0.3, 4.4] | - | - |
| Malawi 2015-16 | 2.7 [1.4, 5.4] | 1.7 [1.2, 2.4] | 1.8 [1.0, 3.2] | 0.4 [0.1, 1.0] | -2.3 | 0.3136 |
| Mali 2018 | 1.5 [1.0, 2.4] | 2.5 [1.1, 5.6] | 1.5 [0.6, 3.7] | 0.0 [0.0, 0.0] | -1.5 | 0.6396 |
| Mozambique 2011 | 8.4 [7.0, 10.1] | 7.5 [6.4, 8.8] | 6.5 [4.7, 8.9] | 1.6 [0.2, 11.6] | -6.8 | 0.1887 |
| Namibia 2013 | 0.0 [0.0, 0.0] | 1.7 [0.7, 4.0] | 0.5 [0.2, 1.2] | 0.0 [0.0, 0.0] | 0.0 | 0.2019 |
| Niger 2012^¶^ | 1.6 [1.0, 2.5] | 0.5 [0.2, 1.4] | 0.9 [0.2, 2.9] | - | - | - |
| Nigeria 2018 | 2.3 [1.7, 3.2] | 2.3 [1.4, 3.8] | 1.9 [1.4, 2.7] | 1.2 [0.5, 2.9] | -1.1 | 0.4841 |
| Rwanda 2014-15 | 3.2 [1.7, 6.0] | 2.0 [1.4, 2.8] | 2.6 [1.2, 5.6] | 0.0 [0.0, 0.0] | -3.2 | 0.4029 |
| STP 2008-09^¶^ | 18.1 [7.1, 38.8] | 7.0 [5.0, 9.8] | 10.0 [5.8, 16.7] | - | - | - |
| Senegal 2010-11^¶^ | 3.2 [2.2, 4.6] | 1.7 [0.8, 3.8] | 2.4 [0.4, 12.4] | - | - | - |
| Sierra Leone 2013 | 6.7 [5.5, 8.1] | 3.5 [2.0, 5.9] | 6.0 [3.9, 9.0] | 4.6 [0.8, 23.1] | -2.1 | 0.1827 |
| South Africa 2016^¶^ | - | 6.8 [3.1, 14.3] | 5.8 [3.7, 8.7] | 0.7 [0.1, 5.4] | - | - |
| Tanzania 2015-16 | 2.6 [1.4, 4.9] | 2.3 [1.8, 3.0] | 2.8 [1.7, 4.4] | 1.3 [0.3, 5.7] | -1.3 | 0.8024 |
| Togo 2013-14^¶^ | 0.7 [0.3, 1.7] | 1.1 [0.5, 2.1] | 2.9 [1.5, 5.5] | - | - | - |
| Uganda 2016 | 1.8 [0.7, 4.6] | 2.0 [1.4, 3.0] | 2.5 [1.3, 4.9] | 2.2 [0.8, 6.2] | 0.4 | 0.9065 |
| Zimbabwe 2015 | 1.6 [0.2, 12.5] | 1.3 [0.7, 2.3] | 1.1 [0.7, 1.5] | 3.9 [1.5, 9.7] | 2.3 | 0.0279 |
| **EMRO** | **-** | **-** | **-** | **-** | **-** |  |
| Egypt 2014 | 5.4 [3.5, 8.2] | 7.2 [4.2, 12.0] | 5.3 [4.2, 6.7] | 6.6 [4.3, 10.1] | 1.2 | 0.6411 |
| Yemen 2013^§^ | - | - | - | - | - | - |
| **EURO** | **-** | **-** | **-** | **-** | **-** |  |
| Albania 2017-18^¶^ | - | 7.3 [5.2, 10.1] | 2.2 [1.1, 4.5] | 3.9 [1.8, 8.1] | - | - |
| Armenia 2015-16^¶^ | - | 1.9 [0.2, 13.3] | 2.4 [1.4, 4.3] | 1.5 [0.7, 3.1] | - | - |
| Azerbaijan 2006^¶^ | - | - | 9.1 [7.6, 10.9] | 4.4 [3.6, 5.4] | - | - |
| Kyrgyz Republic 2012^¶^ | - | - | 6.6 [5.0, 8.6] | 5.1 [3.6, 7.1] | - | - |
| Moldova 2005^¶^ | - | - | 3.9 [2.7, 5.6] | 3.8 [2.4, 6.0] | - | - |
| Tajikistan 2017 | 1.0 [0.1, 7.2] | 2.3 [0.8, 6.1] | 2.5 [1.9, 3.3] | 1.9 [1.1, 3.4] | 0.9 | 0.6642 |
| **PAHO** | **1.5 [0.8, 2.2]** | **1.7 [1.0, 2.5]** | **1.7 [1.1, 2.3]** | **2.2 [1.6, 2.8]** | **0.7** |  |
| Bolivia 2008 | 7.0 [2.5, 17.8] | 6.6 [4.9, 8.9] | 4.0 [2.5, 6.5] | 2.6 [1.2, 5.5] | -4.4 | 0.0659 |
| Guatemala 2014-15 | 1.0 [0.6, 1.7] | 1.0 [0.7, 1.5] | 0.6 [0.4, 1.1] | 1.8 [0.7, 4.1] | 0.8 | 0.2345 |
| Guyana 2009 | 3.8 [0.4, 27.6] | 1.7 [0.5, 6.0] | 3.1 [1.6, 5.8] | 2.1 [0.3, 14.2] | -1.7 | 0.8108 |
| Haiti 2016-17 | 2.9 [1.5, 5.6] | 1.9 [1.2, 3.1] | 2.4 [1.5, 3.8] | 2.0 [0.6, 6.3] | -0.9 | 0.7323 |
| Honduras 2011-12 | 1.1 [0.3, 3.9] | 0.5 [0.3, 0.8] | 0.9 [0.6, 1.6] | 3.3 [1.5, 7.1] | 2.2 | 0.0001 |
| Peru 2012 | 0.8 [0.2, 3.8] | 1.2 [0.8, 1.9] | 1.3 [0.8, 2.0] | 3.1 [2.1, 4.7] | 2.3 | 0.0010 |
| **SEARO** | **1.6 [0.3, 2.8]** | **1.3 [0.4, 2.2]** | **1.8 [0.9, 2.6]** | **2.0 [1.2, 2.8]** | **0.4** |  |
| India 2015-16 | 1.8 [1.7, 2.0] | 1.7 [1.5, 2.0] | 1.8 [1.7, 2.0] | 2.1 [1.8, 2.6] | 0.3 | 0.1959 |
| Maldives 2016-17 | 2.8 [0.3, 21.9] | 3.0 [1.8, 5.0] | 3.7 [2.5, 5.7] | 3.5 [1.6, 7.2] | 0.7 | 0.9100 |
| Myanmar 2015-16 | 0.1 [0.0, 0.7] | 0.6 [0.2, 1.3] | 0.7 [0.3, 1.7] | 1.9 [0.6, 6.1] | 1.8 | 0.0750 |
| Nepal 2016 | 0.0 [0.0, 0.0] | 0.4 [0.0, 2.7] | 0.5 [0.2, 1.4] | 1.1 [0.4, 3.4] | 1.1 | 0.1324 |
| Timor-Leste 2016 | 2.2 [1.0, 4.7] | 1.1 [0.3, 4.1] | 2.5 [1.3, 4.8] | 4.2 [1.2, 13.5] | 2.0 | 0.4564 |
| **WPRO** | **-** | **-** | **-** | **-** | **-** |  |
| Cambodia 2014 | 1.0 [0.4, 2.6] | 1.2 [0.7, 1.9] | 1.5 [0.8, 2.7] | 2.5 [0.3, 16.3] | 1.5 | 0.7379 |
| **Overall pooled prevalence** | **2.4 [2.0, 2.9]** | **2.3 [1.9, 2.7]** | **2.3 [2.0, 2.7]** | **2.1 [1.8, 2.4]** | **-0.3** |  |

AFRO, African region; DRC, Democratic Republic of the Congo; STP, Sao Tome and Principe; EMRO, Eastern Mediterranean region; EURO, European region; PAHO, Americas region; SEARO, Southeast Asian region; WPRO, Western Pacific region.

*Values are percentages and 95% CIs; estimates account for survey design. Regional estimates are pooled prevalences and 95% CIs, calculated with the available data from countries within that region. The WPRO region only had one country with available data (Cambodia), thus the regional prevalence was not calculated.

† Gaps are expressed in percentage points and indicate the difference between the highest and lowest education level (E4-E1).

^‡^ p-values <0.05 indicate significant differences in the distribution of concurrent overweight/obesity and anaemia by education level.

^§^ Yemen has missing data on education level, and thus, the stratified estimates could not be calculated.

^¶^ Estimates for certain categories are missing due to sample size <25.

**Supplemental Table 9.** Households with anaemia among mothers and overweight/obesity among children by area of residence.

|  | **Area of residence^*^** | | | |
| --- | --- | --- | --- | --- |
| **Country and survey year** | **Urban** | **Rural** | **Gap**^†^ | **p-value**^‡^ |
| **AFRO** | **2.8 [2.2, 3.3]** | **2.8 [2.3, 3.3]** | **0.0** |  |
| Benin 2017-18 | 0.9 [0.5, 1.5] | 1.2 [0.8, 1.8] | -0.3 | 0.3085 |
| Burkina Faso 2010 | 2.4 [1.4, 4.1] | 2.5 [1.8, 3.3] | -0.1 | 0.9119 |
| Burundi 2016-17 | 0.3 [0.1, 1.4] | 0.9 [0.6, 1.4] | -0.6 | 0.1317 |
| Cameroon 2011 | 3.6 [2.7, 4.8] | 3.8 [2.8, 5.3] | -0.2 | 0.7448 |
| Congo 2011-12 | 2.9 [1.8, 4.6] | 1.9 [1.3, 2.6] | 1.0 | 0.1306 |
| Cote d'Ivoire 2011-12 | 2.8 [1.4, 5.6] | 2.6 [1.8, 3.9] | 0.2 | 0.8428 |
| DRC 2013-14 | 2.2 [1.4, 3.4] | 3.7 [2.8, 5.0] | -1.5 | 0.0401 |
| Eswatini 2006-07 | 6.7 [4.3, 10.4] | 3.8 [2.7, 5.2] | 2.9 | 0.0347 |
| Ethiopia 2016 | 1.4 [0.7, 3.0] | 1.2 [0.7, 2.1] | 0.2 | 0.7169 |
| Gabon 2012 | 7.0 [5.0, 9.9] | 7.2 [5.0, 10.1] | -0.2 | 0.9353 |
| Gambia 2013 | 3.0 [1.5, 5.9] | 3.3 [2.2, 5.0] | -0.3 | 0.8357 |
| Ghana 2014 | 1.6 [0.8, 3.1] | 1.6 [0.9, 2.7] | 0.0 | 0.9597 |
| Guinea 2018 | 3.3 [2.1, 5.4] | 2.8 [2.0, 3.8] | 0.5 | 0.5124 |
| Lesotho 2014 | 1.7 [0.4, 6.8] | 2.5 [1.6, 4.0] | -0.8 | 0.5877 |
| Malawi 2015-16 | 1.2 [0.4, 3.3] | 1.9 [1.4, 2.5] | -0.7 | 0.3981 |
| Mali 2018 | 1.7 [0.8, 3.4] | 1.6 [1.1, 2.5] | 0.1 | 0.9006 |
| Mozambique 2011 | 6.8 [5.3, 8.5] | 8.0 [7.0, 9.2] | -1.2 | 0.2122 |
| Namibia 2013 | 0.4 [0.1, 1.3] | 0.9 [0.4, 1.9] | -0.5 | 0.2114 |
| Niger 2012 | 3.1 [1.5, 6.2] | 1.2 [0.7, 2.0] | 1.9 | 0.0226 |
| Nigeria 2018 | 1.7 [1.2, 2.6] | 2.4 [1.8, 3.1] | -0.7 | 0.1992 |
| Rwanda 2014-15 | 1.3 [0.6, 2.9] | 2.4 [1.8, 3.3] | -1.1 | 0.1484 |
| STP 2008-09 | 8.9 [5.7, 13.8] | 7.9 [5.4, 11.5] | 1.0 | 0.6938 |
| Senegal 2010-11 | 2.8 [1.5, 5.1] | 2.8 [1.9, 3.9] | 0.0 | 0.9920 |
| Sierra Leone 2013 | 5.0 [3.6, 6.9] | 6.5 [5.3, 7.9] | -1.5 | 0.1702 |
| South Africa 2016 | 4.8 [2.6, 8.8] | 5.9 [3.9, 8.9] | -1.1 | 0.5876 |
| Tanzania 2015-16 | 2.2 [1.4, 3.3] | 2.5 [1.9, 3.3] | -0.3 | 0.5675 |
| Togo 2013-14 | 1.8 [0.9, 3.5] | 1.2 [0.6, 2.1] | 0.6 | 0.3263 |
| Uganda 2016 | 2.0 [1.0, 4.0] | 2.2 [1.6, 3.0] | -0.2 | 0.8191 |
| Zimbabwe 2015 | 2.1 [1.4, 3.3] | 0.9 [0.6, 1.4] | 1.2 | 0.0067 |
| **EMRO** | **3.9 [3.0, 4.8]** | **4.1 [3.4, 4.7]** | **-0.2** |  |
| Egypt 2014 | 7.5 [5.8, 9.7] | 4.8 [3.7, 6.2] | 2.7 | 0.0143 |
| Yemen 2013 | 1.8 [1.0, 3.4] | 4.3 [3.0, 6.1] | -2.5 | 0.0170 |
| **EURO** | **4.2 [2.6, 5.9]** | **4.5 [3.0, 6.0]** | **-0.3** |  |
| Albania 2017-18 | 5.6 [3.5, 8.9] | 4.6 [3.3, 6.4] | 1.0 | 0.4877 |
| Armenia 2015-16 | 1.4 [0.7, 2.8] | 2.6 [1.5, 4.4] | -1.2 | 0.1689 |
| Azerbaijan 2006 | 8.2 [6.0, 11.1] | 8.6 [6.7, 10.9] | -0.4 | 0.8251 |
| Kyrgyz Republic 2012 | 6.2 [4.1, 9.3] | 5.7 [4.5, 7.2] | 0.5 | 0.7233 |
| Moldova 2005 | 3.1 [1.8, 5.4] | 4.3 [2.8, 6.6] | -1.2 | 0.3590 |
| Tajikistan 2017 | 2.9 [2.0, 4.3] | 2.2 [1.7, 3.0] | 0.7 | 0.2907 |
| **PAHO** | **1.9 [1.3, 2.5]** | **1.8 [1.1, 2.5]** | **0.1** |  |
| Bolivia 2008 | 4.2 [2.9, 5.9] | 6.7 [4.9, 9.1] | -2.5 | 0.0433 |
| Guatemala 2014-15 | 0.9 [0.6, 1.4] | 0.9 [0.7, 1.3] | 0.0 | 0.9157 |
| Guyana 2009 | 3.3 [1.4, 7.6] | 2.6 [1.3, 5.0] | 0.7 | 0.6609 |
| Haiti 2016-17 | 2.7 [1.7, 4.2] | 2.1 [1.4, 3.1] | 0.6 | 0.4195 |
| Honduras 2011-12 | 1.3 [0.9, 1.9] | 0.5 [0.3, 0.7] | 0.8 | 0.0005 |
| Peru 2012 | 1.9 [1.4, 2.6] | 1.4 [1.0, 2.1] | 0.5 | 0.2281 |
| **SEARO** | **1.6 [0.6, 2.5]** | **1.7 [0.8, 2.7]** | **-0.1** |  |
| India 2015-16 | 2.1 [1.8, 2.3] | 1.8 [1.7, 1.9] | 0.3 | 0.0234 |
| Maldives 2016-17 | 3.3 [1.3, 8.3] | 3.6 [2.8, 4.6] | -0.3 | 0.8730 |
| Myanmar 2015-16 | 1.6 [0.8, 3.2] | 0.4 [0.2, 0.8] | 1.2 | 0.0031 |
| Nepal 2016 | 0.3 [0.1, 0.9] | 0.5 [0.2, 1.2] | -0.2 | 0.4745 |
| Timor-Leste 2016 | 3.0 [1.3, 6.9] | 2.0 [1.3, 3.3] | 1.0 | 0.4237 |
| **WPRO** | **-** | **-** | **-** |  |
| Cambodia 2014 | 1.8 [0.8, 3.8] | 1.2 [0.8, 1.8] | 0.6 | 0.3737 |
| **Overall pooled prevalence** | **2.7 [2.3, 3.0]** | **2.7 [2.4, 3.1]** | **0.0** |  |

AFRO, African region; DRC, Democratic Republic of the Congo; STP, Sao Tome and Principe; EMRO, Eastern Mediterranean region; EURO, European region; PAHO, Americas region; SEARO, Southeast Asian region; WPRO, Western Pacific region.

*Values are percentages and 95% CIs; estimates account for survey design. Regional estimates are pooled prevalences and 95% CIs, calculated with the available data from countries within that region. The WPRO region only had one country with available data (Cambodia), thus the regional prevalence was not calculated.

† Gaps are expressed in percentage points and indicate the difference between urban and rural areas (urban-rural).

^‡^ p-values <0.05 indicate significant differences in the distribution of concurrent overweight/obesity and anaemia by area of residence.
